# New Self-assembled Supramolecular Bowls as Potent Anticancer Agents for Human Hepatocellular Carcinoma

**DOI:** 10.1038/s41598-018-36755-9

**Published:** 2019-01-18

**Authors:** Hae Seong Song, Young Ho Song, Nem Singh, Hyunuk Kim, Hyelin Jeon, Inhye Kim, Se Chan Kang, Ki-Whan Chi

**Affiliations:** 1 Department of Oriental Medicine Biotechnology, College of Life Sciences, Kyung Hee University, Yongin, 17104 Republic of Korea; 2 Department of Chemistry, University of Ulsan, Ulsan, 44610 Republic of Korea; 3 Convergence Materials Laboratory, Korea Institute of Energy Research, Daejeon, 28119 Republic of Korea

## Abstract

We report herein on the design, synthesis and biological activity of Ru-based self-assembled supramolecular bowls as a potent anticancer therapeutic in human hepatocellular cancer. The potent complex induces production of reactive oxygen species (ROS) by higher fatty acid β-oxidation and down-regulation of glucose transporter-mediated pyruvate dehydrogenase kinase 1 via reduced hypoxia-inducible factor 1α. Also, overexpressed acetyl-CoA activates the tricarboxylic acid cycle and the electron transport system and induces hypergeneration of ROS. Finally, ROS overexpressed through this pathway leads to apoptosis. Furthermore, we demonstrate that the naphthalene derived molecular bowl activates classical apoptosis via crosstalk between the extrinsic and intrinsic signal pathway. Our work into the mechanism of Ru-based self-assembled supramolecular bowls can provide valuable insight into the potential for use as a promising anticancer agent.

## Introduction

Hepatocellular carcinoma (HCC) is currently the fifth most common cancer in the world and the third most common cancer that leads to death^1^. Therefore, development of new therapies against HCC is important, and many drugs have recently been developed^2,3^. However, the high incidence of intrahepatic recurrence remains a major challenge in HCC therapy^4,5^.

Self-assembly is a ubiquitous phenomenon in natural systems that is involved in core processes in a living organism including protein synthesis and DNA formation. Due to efforts to mimic natural processes in the laboratory, research on self-assembly has grown immensely in the past few decades^6–9^. Of the various self-assembly methods, coordination-driven self-assembly is an efficient method for constructing supramolecular architecture with desired shapes for promising applications in molecular recognition, separation, catalysis, encapsulation of guests and biological systems^10–17^. Some metals such as Pd, Pt and Ru are used as metal centers of coordination-driven self-assembly. Among them, the biological activity of Ru complexes is unknown^18,19^. But favorable results with Ru-based self-assembled architectures as anticancer compounds have been reported by our study^20–24^. The amide functional group plays an important role in the biochemical processes of nature and is a versatile precursor to many other functional groups. Specifically, benzamide derivatives are promising building blocks for bioactive compounds since they are found in various well-known drugs^25–28^. Therefore, a new dipyridyl benzamide ligand was synthesized and used for self-assembly in this work.

One of the emerging hallmarks of human cancer is the reprogramming of cellular energy metabolism to induce tumorigenesis, progression and survival^29^. Human carcinoma is associated with increased cellular glucose uptake and an enhanced metabolic state. Warburg first reported that tumor cells increased aerobic glycolysis rather than mitochondrial oxidative phosphorylation (OXPHOS), known as the “Warburg effect”^30^. However, many researchers have sought to understand the reprogramming of this cellular mechanism over the past 2 decades^31^. Recent studies have suggested an equally meaningful role of the tricarboxylic acid (TCA) cycle and OXPHOS in tumor progression^32,33^. Therefore, we proposed that the new dipyridyl benzamide ligand would play a role in the glycolysis or mitochondrial metabolism, leading to inhibition of tumor survival and progression in HCC.

Hypoxia-inducible factor-1α (HIF-1α) is an important transcription factor in the transport of glucose metabolites from the mitochondria and regulation of cellular response to hypoxia^34,35^. HIF-1α is significantly overexpressed in patients with HCC^36^. The apparent overexpression of HIF-1α suggests a potential marker for development of a therapeutic agent in HCC^37^.Also, according to another report, HIF-1α activates PDHK-1, which inhibits pyruvate dehydrogenase (PDH), an enzyme that converts pyruvate to acetyl-CoA. When HIF-1α decreases, acetyl-CoA is induced and overexpressed acetyl-CoA activates the TCA cycle, leading to the production of electron donors NADH, FADH_2_ and ROS^34,38^. Apoptosis is induced through the release of cytochrome c into the cytoplasm by lowering the potential of the mitochondrial membrane^34^. Furthermore, hypergeneration of ROS induces apoptosis of HCC by causing depolarization of mitochondria and activation of caspase-9. Therefore, hypoxia induced by cancer causes apoptosis resistance due to this mechanism.

Classical apoptosis is initiated by two major signaling pathways^39^. In the extrinsic pathway, the Fas ligand binds to the Fas receptor, resulting in activation of caspase-8 via multi-signal transduction^40^. The intrinsic pathway is initiated by non-receptor-mediated stimuli such as hypoxia, ROS and viral infections. Upon triggering these stimuli, Bax and Bak interact with Bid, resulting in the induction of the mitochondrial permeability transition (MPT) pore and release of cytochrome c to the cytoplasm^41^. Subsequently, cytochrome c recruits the clustering of procaspase-9 called the apoptosome, which leads to activation of caspase-9 and effector caspases including caspases-3, -6 and -7^42^. Once caspase-3 is activated, poly (ADP-ribose) polymerase (PARP) is inactivated by cleavage into 89-kDa and 24-kDa fragments, leading to DNA fragmentation and execution of apoptosis^43^. These distinguished pathways are connected by caspase-8-mediated cleavage of Bid, resulting in “cross-talk”^44^.

Herein, we report the design of a new dipyridyl benzamide ligand and the synthesis of molecular bowls using arene-Ru acceptors. This article also shows the molecular mechanisms by which the naphthalene derived molecular bowl **8** (MB**8)** inhibits the survival and proliferation of HCC via the suppression of HIF-1α and the activation of cross-talk between the extrinsic and intrinsic apoptosis pathway.

## Results and Discussion

### Dipyridyl benzamide donor 1

The new dipyridyl benzamide ligand 1 was synthesized by Sonagashira coupling of 3,5-dibromobenzamide and fully characterized by ^1^H^13^,C NMR and high-resolution mass spectroscopy. (Figs S1 and S2, Supplementary Information). Yield: 63%. Mp: 226–228 °C. Anal. calcd. for C_21_H_13_N_3_O: C, 78.00; H, 4.05; N, 13.00. Found: C, 78.32; H, 4.22; N, 12.90. ^1^H NMR (400 MHz, CDCl_3_) δ 8.77 (s, 2 H), 8.58 (d, *J* = 3.7 Hz, 2 H), 7.96 (s, 2 H), 7.89–7.78 (m, 3 H), 7.37–7.29 (m, 2 H). ^13^C NMR (101 MHz, CDCl_3_) δ 167.48, 152.30, 149.10, 139.05, 137.67, 134.43, 130.80, 124.01, 123.48, 120.05, 90.89, 87.92.HR-MS m/z: 323.1057 (Calcd for C_21_H_13_N_3_O, 323.1059).

### Synthesis and characterization of molecular bowls 6–9

**Figure 1:**
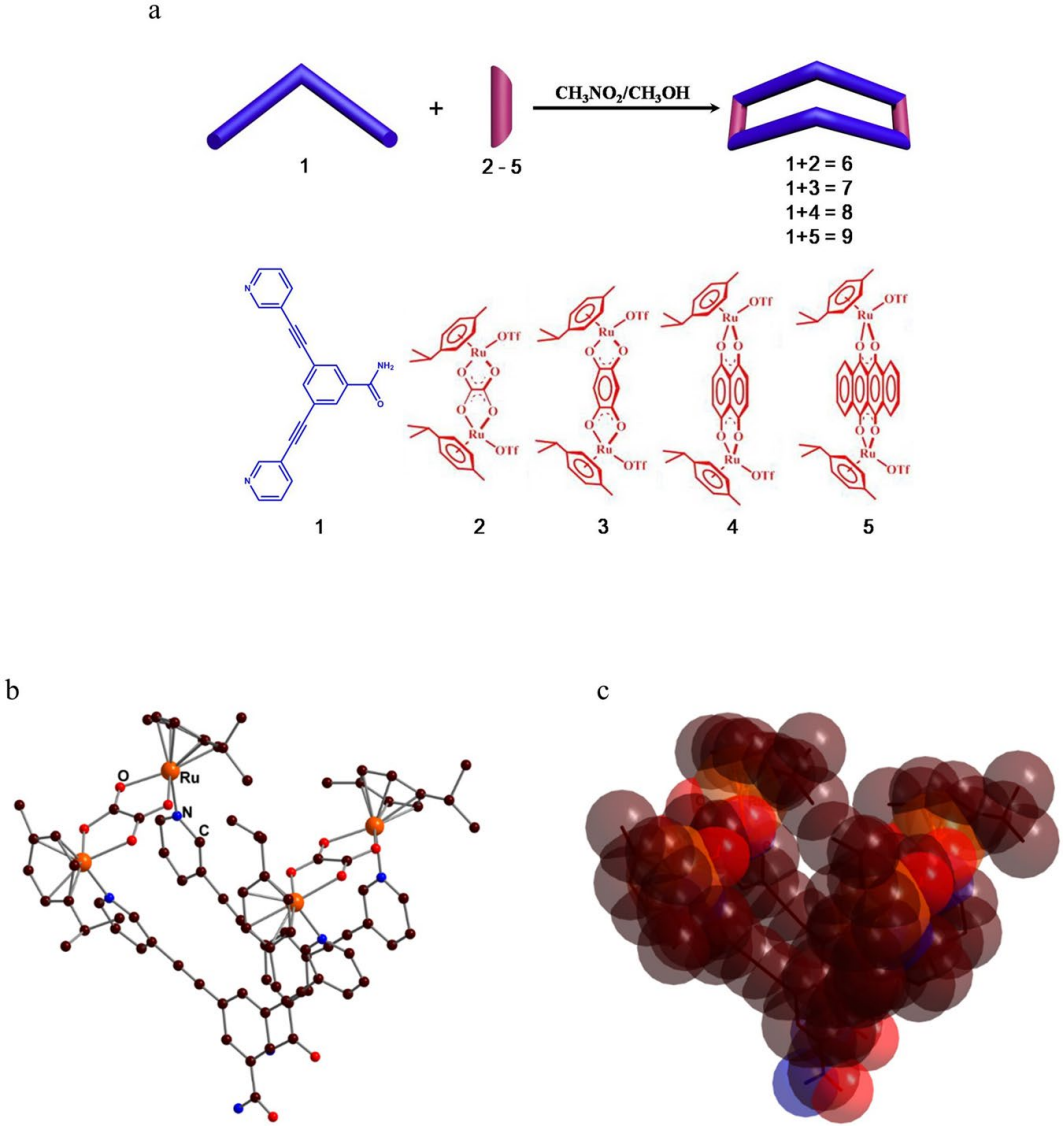
Structure of molecular bowls. (**a**) Coordination-driven self-assembly molecular bowls 6–9. (**b**) X-ray crystal structure of 6 in ball and stick model, (**c**) space-filling representation superimposing capped stick. Counter-anions and hydrogen atoms are omitted for clarity.

The new dipyridyl benzamide ligand **1** was synthesized by Sonagashira coupling of 3,5-dibromobenzamide and fully characterized by ^1^H^13^,C NMR and high-resolution mass spectroscopy. The pure dipyridyl benzamide ligand **1** with proper dinuclear Ru(II) acceptors **2**–**5** was used for self-assembly of molecular bowls **6**–**9** in CH_3_NO_2_/CH_3_OH (1/1) cosolvent (Fig. 1). The reaction mixtures were stirred at room temperature for 6 h to obtain clear solutions. Molecular bowls **6**–**9** were purified by precipitation and filtration and then fully characterized by ^1^H and ^13^C NMR, ESI-MS, and elemental analysis. The solid-state structure of complex **6** was determined by single-crystal X-ray analysis. The ^1^H NMR peaks associated with building blocks were shifted after self-assembly reaction in CD_3_OD/CD_3_NO_2_ (1/1) cosolvent. This result indicated the formation of molecular bowls **6**–**9** via metal-ligand coordination bonding (Figures S3–S10). The α-pyridyl protons of ligand **1** shifted upfield by 0.2–0.6 ppm on complexation with acceptors **2**–**5**. The formation of molecular bowls **6**–**9** was further supported by ESI-MS analysis. The isotopic distribution peaks for molecular bowls **6**, **7**, **8** and **9** observed at m/z 638.09, 671.45, 704.80 and 771.51, respectively, corresponded to [M–3OTf]^3+^ (Figure S11). These peaks matched well with the theoretical distributions. Single-crystal X-ray diffraction (SCXRD) analysis of **6** unambiguously confirmed its molecular structure (Fig. 1 and S12). Slow vapor diffusion of diethyl ether into the nitromethane/methanol solution of **6** yielded light yellow single crystals suitable for SCXRD analysis. The structure of macrocycle **6** was refined in the triclinic Pspace group and showed a bowl-shaped geometry. Interestingly, the benzamide moieties interacted with displaced π−π stacking at a distance of 3.671^1^ Å; thus, the stacked semicircles resulting in a bowl-shaped architecture.

### Molecular bowl 6

Molecular bowl **6** was self-assembled via the general procedure described above using benzamide donor **1** (0.97 mg, 3.0 µmol) and acceptor **2** (2.57 mg, 3.0 µmol). The bright-yellow colored crystalline powder was characterized as **6**. Isolated yield: 93%. Mp: 236–237 °C (dec.). Anal. calcd. for C_90_H_82_F_12_N_6_O_22_Ru_4_S_4_: C, 45.80; H, 3.50; N, 3.56. Found: C, 46.07; H, 3.67; N, 3.32. ^1^H NMR (300 MHz, CD_3_NO_2_/CD_3_OD) δ 8.22 (d, *J* = 1.7 Hz, 2 H), 8.06 (dt, *J* = 8.0, 1.7 Hz, 2 H), 7.94 (dd, *J* = 5.8, 1.7 Hz, 2 H), 7.82 (d, *J* = 1.5 Hz, 2 H), 7.65 (t, *J* = 1.5 Hz, 1 H), 7.38 (dd, *J* = 8.0, 5.8 Hz, 2 H), 5.95 (br, 4 H), 5.79 (d, *J* = 6.0 Hz, 4 H), 2.95–2.81 (m, *J* = 7.0 Hz, 2 H), 2.24 (s, 6 H), 1.37 (d, *J* = 7.0 Hz, 12 H). ^13^C NMR (101 MHz, MeOD) δ 172.35, 169.13, 155.40, 152.85, 143.83, 138.65, 135.81, 132.83, 127.68, 124.03, 123.95, 104.16, 99.29, 95.04, 86.00, 83.59, 83.19, 82.60, 32.61, 22.65, 18.21.ESI-MS for C_87_H_82_F_3_N_6_O_13_Ru_4_S: calcd., 638.06 [**6**-3OTf]^+3^; observed, 638.09.

### Molecular bowl 7

Molecular bowl **7** was self-assembled via the general procedure described above using benzamide donor **1** (0.97 mg, 3.0 µmol) and acceptor **3** (2.72 mg, 3.0 µmol). The dark-red colored crystalline powder was characterized as **7**. Isolated yield: 95%. Mp: 235–237 °C (dec.). Anal. calcd. for C_98_H_86_F_12_N_6_O_22_Ru_4_S_4_: C, 47.84; H, 3.52; N, 3.42. Found: C, 48.11; H, 3.73; N, 3.15. ^1^H NMR (300 MHz, CD_3_NO_2_/CD_3_OD) δ 8.34 (d, *J* = 1.8 Hz, 2 H), 8.16–8.08 (m, 4 H), 7.86 (d, *J* = 1.5 Hz, 2 H), 7.69 (t, *J* = 1.5 Hz, 1 H), 7.43 (dd, *J* = 7.9, 5.8 Hz, 2 H), 6.04 (d, *J* = 6.3 Hz, 4 H), 5.82 (d, *J* = 6.3 Hz, 4 H), 5.77 (s, 2 H), 2.96–2.85 (m, *J* = 6.9 Hz, 2 H), 2.21 (s, 6 H), 1.35 (d, *J* = 6.9 Hz, 12 H). ^13^C NMR (101 MHz, MeOD) δ 183.41, 167.73, 154.60, 151.93, 141.75, 134.37, 131.22, 125.68, 122.34, 122.25, 122.18, 122.07, 118.90, 104.08, 98.91, 92.80, 84.29, 83.31, 81.59, 31.21, 21.16, 16.81.ESI-MS for C_95_H_86_F_3_N_6_O_13_Ru_4_S: calcd., 671.41 [**7**-3OTf]^+3^; observed, 671.45.

### Molecular bowl 8

Molecular bowl **8** was self-assembled via the general procedure described above using benzamide donor **1** (0.97 mg, 3.0 µmol) and acceptor **4** (2.87 mg, 3.0 µmol). The greenish colored crystalline powder was characterized as **8**. Isolated yield: 93%. Mp: 238–240 °C (dec.). Anal. calcd. for C_106_H_90_F_12_N_6_O_22_Ru_4_S_4_: C, 49.72; H, 3.54; N, 3.28. Found: C, 50.03; H, 3.72; N, 3.15. ^1^H NMR (300 MHz, CD_3_NO_2_/CD_3_OD) δ 8.50 (d, *J* = 1.6 Hz, 2 H), 8.31 (dd, *J* = 5.8, 1.6 Hz, 2 H), 8.02 (dt, *J* = 7.8, 1.6 Hz, 2 H), 7.86 (d, *J* = 1.5 Hz, 2 H), 7.67 (t, *J* = 1.5 Hz, 1 H), 7.37 (dd, *J* = 7.8, 5.8 Hz, 2 H), 7.23 (s, 4 H), 5.82 (d, *J* = 6.3 Hz, 4 H), 5.59 (d, *J* = 6.3 Hz, 4 H), 2.96–2.81 (m, *J* = 7.0 Hz, 2 H), 2.14 (s, 6 H), 1.34 (d, *J* = 7.0 Hz, 12 H). ^13^C NMR (75 MHz, CD_3_NO_2_/CD_3_OD) δ 171.96, 155.07, 152.57, 148.93, 142.70, 138.51, 138.06, 135.77, 132.44, 126.56, 123.78, 122.94, 104.96, 101.27, 101.04, 93.27, 90.93, 86.04, 85.41, 83.87, 31.92, 22.33, 17.37.ESI-MS for C_103_H_90_F_3_N_6_O_13_Ru_4_S: calcd., 704.75 [**8**-3OTf]^+3^; observed, 704.80.

### Molecular bowl 9

Molecular bowl **9** was self-assembled via the general procedure described above using benzamide donor **1** (0.97 mg, 3.0 µmol) and acceptor **5** (3.17 mg, 3.0 µmol). The dark-green colored crystalline powder was characterized as **9**. Isolated yield: 91%. Mp: 242–243 °C (dec.). Anal. calcd. for C_122_H_98_F_12_N_6_O_22_Ru_4_S_4_: C, 53.08; H, 3.58; N, 3.04. Found: C, 53.32; H, 3.71; N, 3.12. ^1^H NMR (300 MHz, CD_3_NO_2_/CD_3_OD) δ 8.70 (dd, *J* = 6.1, 3.3 Hz, 4 H), 8.56 (d, *J* = 1.8 Hz, 2 H), 8.31 (dd, *J* = 5.7, 1.8 Hz, 2 H), 7.96 (dd, *J* = 6.1, 3.3 Hz, 4 H), 7.92 (dt, *J* = 7.8, 1.8 Hz, 2 H), 7.60 (d, *J* = 1.5 Hz, 2 H), 7.30 (t, *J* = 1.5 Hz, 1 H), 7.20 (dd, *J* = 7.8, 5.7 Hz, 2 H), 6.02 (d, *J* = 6.3 Hz, 4 H), 5.76 (d, *J* = 6.3 Hz, 4 H), 3.10–2.98 (m, *J* = 6.9 Hz, 2 H), 2.27 (s, 6 H), 1.39 (d, *J* = 6.9 Hz, 12 H). ^13^C NMR (75 MHz, CD_3_NO_2_/CD_3_OD) δ 170.75, 155.27, 152.54, 142.67, 137.78, 135.70, 135.27, 134.66, 132.59, 128.63, 126.84, 124.28, 123.60, 123.03, 120.06, 108.60, 105.07, 101.47, 93.32, 86.09, 85.77, 83.65, 32.33, 22.84, 18.20.ESI-MS for C_119_H_98_F_3_N_6_O_13_Ru_4_S: calcd., 771.44 [**9**-3OTf]^+3^; observed, 771.51.

### Cytotoxicity of molecular bowl 8 against human cancer cell lines

We first investigated the cytotoxic potential of MB**8** on a variety of human cancer cell lines by determining their IC_50_. The inhibition effect was assessed in AGS, A549, HCT-15, SK-HEP-1 and HepG2 cells, respectively. MB**8** showed the strongest anticancer effect in all of the tested cell lines (Table 1). In particular, MB**8** had the greatest inhibitory effect in another human HCC line, HepG2. Based on these results, we selected the HepG2 hepatic cell line for further analysis of the anticancer potential of MB**8**.

**Table 1:** Screening of molecular bowls inhibitory effect on various cancer cells growth (IC_50_: μM).

Sample	AGS		A549		HCT-15		SK-HEP-1		HepG2	
	24 h	48 h	24 h	24 h	24 h	48 h	24 h	48 h	24 h	48 h
Dipyridyl benzamide donor 1	100	100	100	100	100	100	100	80.02 ± 4.248	100	88.13 ± 2.324
Dinuclear Ru(II) acceptor 2	100	98.33 ± 4.218	100	100	100	100	100	100	100	100
Dinuclear Ru(II) acceptor 3	100	76.08 ± 4.683	100	99.39 ± 5.108	100	100	100	85.63 ± 3.668	100	84.67 ± 5.462
Dinuclear Ru(II) acceptor 4	87.26 ± 4.167	75.22 ± 3.529	100	100	90.63 ± 5.873	79.26 ± 3.438	81.26 ± 4.576	70.27 ± 2.352	82.67 ± 3.442	80.22 ± 2.884
Dinuclear Ru(II) acceptor 5	100	100	100	100	100	68.22 ± 2.287	100	72.25 ± 2.323	100	78.28 ± 2.664
Molecular bowl 6	24.73 ± 4.034	4.14 ± 0.146	100	60.27 ± 9.116	60.27 ± 9.116	64.89 ± 6.744	60.27 ± 9.116	17.87 ± 0.465	63.24 ± 5.287	20.87 ± 2.002
Molecular bowl 7	29.40 ± 1.909	5.32 ± 0.493	100	19.53 ± 4.352	19.53 ± 4.352	38.89 ± 10.87	19.53 ± 4.352	27.87 ± 4.403	18.63 ± 2.116	22.67 ± 1.537
Molecular bowl 8	5.10 ± 0.388	1.39 ± 0.018	100	5.12 ± 0.125	5.12 ± 0.125	1.57 ± 0.029	5.12 ± 0.125	1.56 ± 0.031	1.09 ± 0.019	0.78 ± 0.004
Molecular bowl 9	100	4.35 ± 0.224	100	100	100	13.64 ± 4.776	100	7.79 ± 1.825	84.64 ± 2.014	18.98 ± 1.121
Cisplatin	90.97 ± 4.337	4.84 ± 0.350	100	33.94 ± 1.498	33.94 ± 1.498	30.03 ± 0.215	33.94 ± 1.498	11.70 ± 1.077	20.89 ± 0.892	12.03 ± 2.646

### Molecular bowl 8 and β-oxidation by activating acyl-CoA dehydrogenases

**Figure 2:**
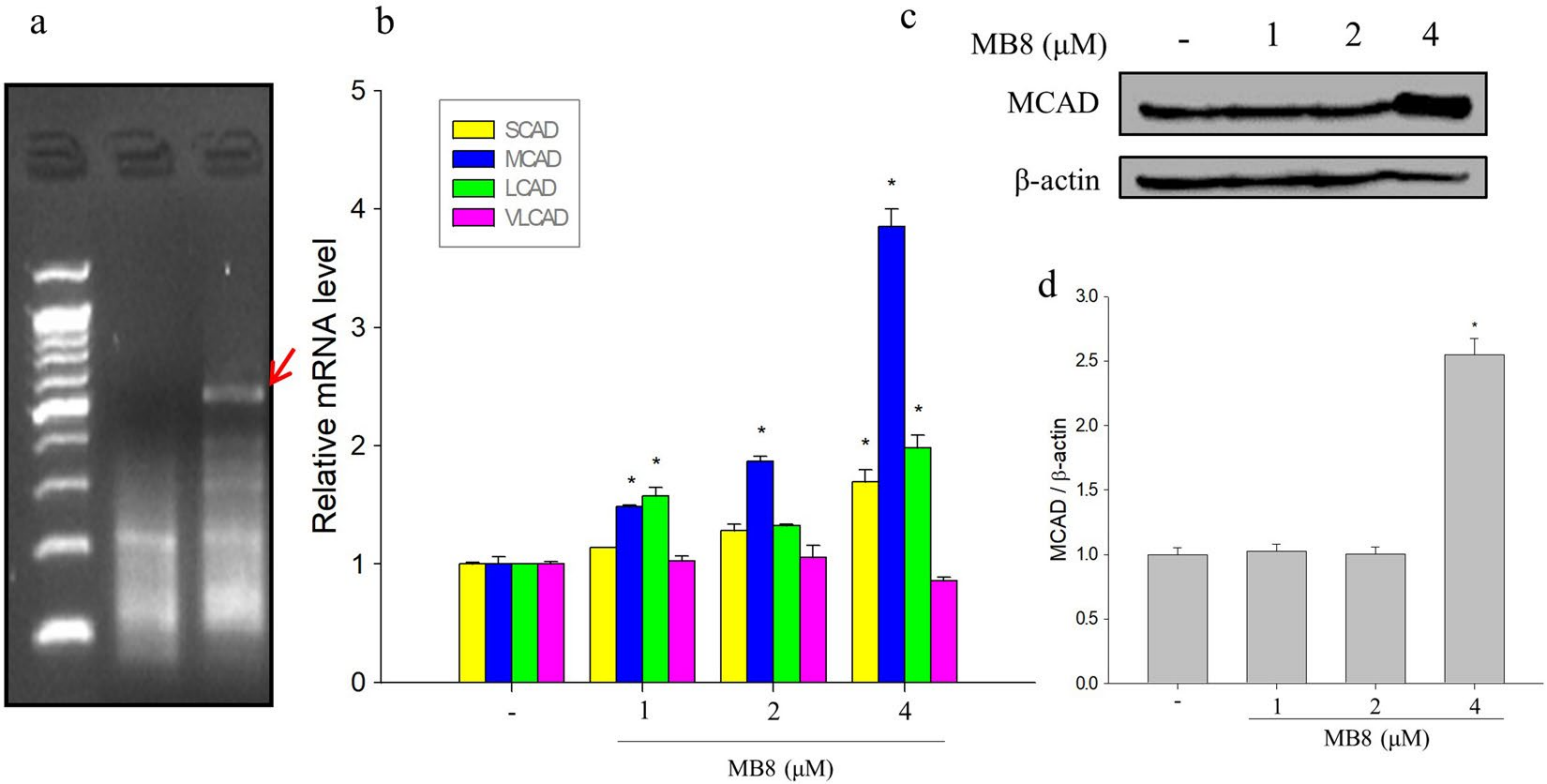
MB**8** induces β-oxidation by activating SCAD, MCAD and LCAD. (**a**) GeneFishing^TM^ DEG screening results. Indicated band was overexpressed by MB**8**. HepG2 cells (2 × 10^5^ cells/well) were seeded on a 6-well plate and incubated for 24 h. After that, MB8 was treated by concentration (1, 2 and 4 μM), for 24 h. (**b**) mRNA expression of genes detected by qRT-PCR. (**c**) The protein level was analyzed by Western blot. (**d**) Densitometric analysis of Western blots is represented as the mean band density. Representative data are shown as the mean ± standard error of the mean (SEM) of each group. *P < 0.05 compared with the control.

To identify genes that are actively expressed by treatment with MB**8**, we compared mRNA expression using ACP-based GeneFishing PCR technology. When we treated MB**8**, 2 bands were increased, and 1 band was decreased in comparison to the control by ACP9 primer (Fig. 2a). As shown in Fig. 2a, the bands of treated MB**8** increased compared to the control (lane 1). We next confirmed the DNA sequence of actively expressed genes via comparison with GenBank (NIH, MD, USA) and then estimated very-long-chain acyl-CoA dehydrogenase (VLCAD), which was related to β-oxidation. There are four distinct acyl-CoA dehydrogenases: short-chain acyl-CoA dehydrogenase (SCAD), medium-chain acyl-CoA dehydrogenase (MCAD), long-chain acyl-CoA dehydrogenase (LCAD) and very-long-chain acyl-CoA dehydrogenase (VLCAD). We performed qRT-PCR to further confirm the β-oxidation-related genes and to determine if VLCAD was involved. Contrary to the expectation, MB**8** significantly increased expression of SCAD, MCAD and LCAD mRNA in comparison with the untreated cells while the expression of VLCAD mRNA did not show a significant change. In particular, MB**8** at concentrations of 1, 2 and 4 µM produced significant up-regulation of MCAD mRNA expression by 1.48, 1.87 and 3.85 times, respectively, compared to the untreated cells (Fig. 2b). Furthermore, MB**8** at a concentration of 4 µM significantly up-regulated the expression of MCAD protein by 2.52-fold compared to untreated cells (Fig. 2c,d). The protein level of MCAD and LCAD was significantly decreased under hypoxia in the cancer cell lines including HepG2, Hep3B and SK-HEP-1, and HIF-1 inhibited MCAD and LCAD, resulting in decreased ROS levels and enhanced tumor proliferation^38^. In this regard, our data indicate that MB**8** induces fatty acid oxidation via the up-regulation of acyl-CoA dehydrogenase, especially MCAD, and then affects HepG2 cancer cell survival.

### Molecular bowl 8 and suppression of HIF-1α and PDHK-1

**Figure 3:**
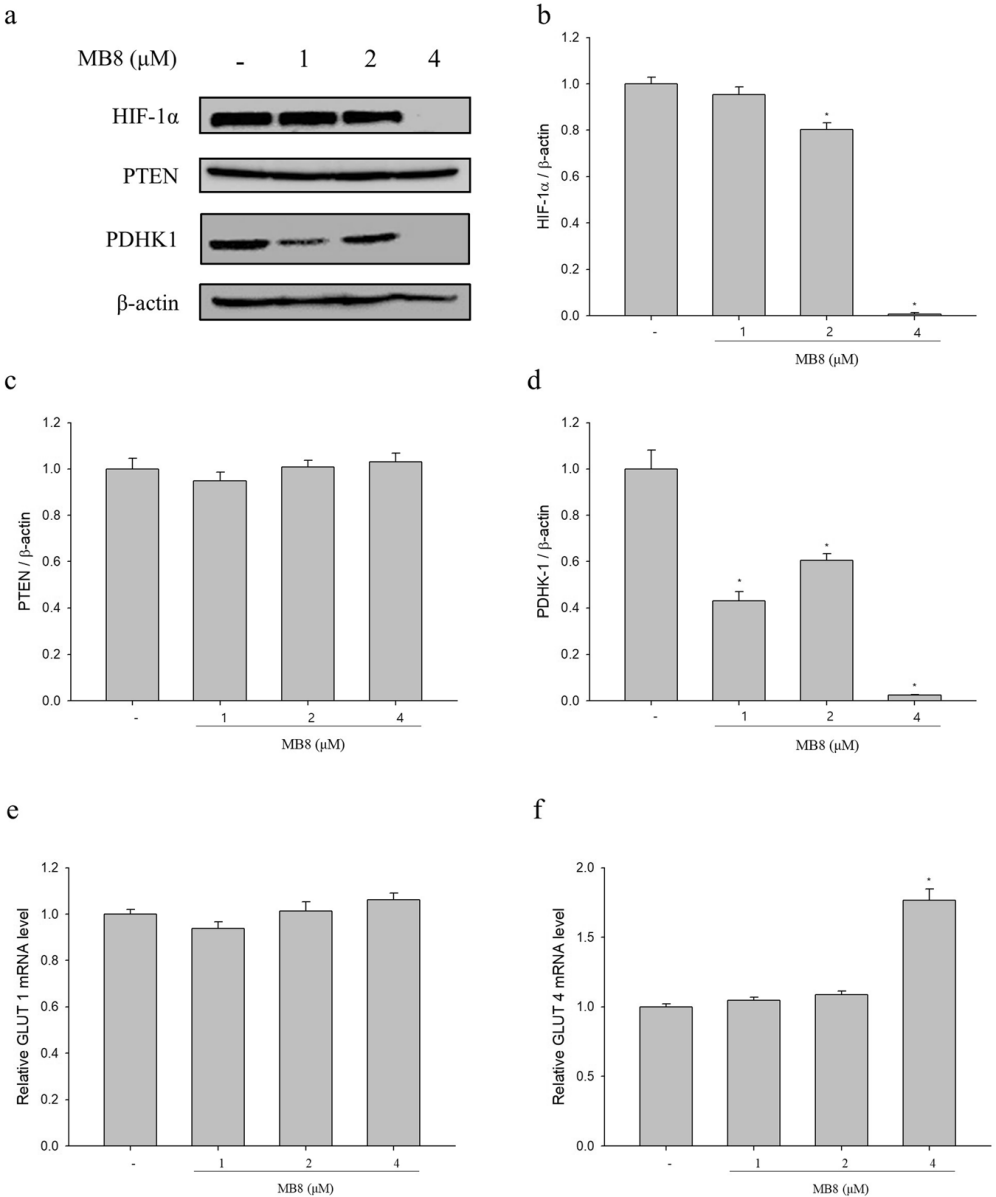
MB**8** suppresses hypoxia by inhibiting the expression of HIF-1α and PDHK-1, while activates glucose transport by altering GLUT4. HepG2 cells (4 × 10^5^ cells/well) were seeded on a 6-well plate and incubated for 24 h. After that, MB8 was treated by concentration (1, 2 and 4 μM), and when it was 24 h. (**a**) The protein levels were analyzed by Western blot. (**b**–**d**) Densitometric analysis of Western blots is represented as the mean band density. (**e**,**f**) mRNA expression of GLUT1 and GLUT4 were detected by qRT-PCR. Representative data are shown as the mean ± standard error of the mean (SEM) of each group. *P < 0.05 compared with the control.

Next, we investigated the level of HIF-1α protein in HepG2 cells treated with MB**8**, which induced acetyl-CoA by acyl-CoA dehydrogenase-mediated β-oxidation or PDHK-1 (Fig. 3a). As shown in Fig. 3b, MB**8** significantly decreased the level of HIF-1α protein in a dose-dependent manner by 0.95-, 0.79-, and 0.002-fold compared with the untreated cells. HIF-1α suppressed the MCAD and LCAD, followed by tumor progression via fatty acid oxidation and PTEN^38^ .Contrary to our expectation, PTEN did not show significant changes from MB**8** treatment at any of the concentrations tested when compared to the untreated cells (Fig. 3c). Accordingly, we assumed that MB**8** should act on cancer cell death via a different route. The HIF-1α is stabilized and translocates to the nucleus under hypoxic conditions, where it dimerizes with HIF-1β and activates the expression of a variety of target genes including pyruvate dehydrogenase kinase-1 (PDHK-1), also known as PDK-1, and glucose transporters during tumorigenesis and cancer progression^45,46^. PDHK-1 is a key rate-limiting enzyme for pyruvate conversion to acetyl-coA, after which it enters into the TCA cycle. Under hypoxic conditions, the conversion of pyruvate to acetyl-coA is decreased by PDHK-1-mediated inhibition of PDH, resulting in the reduced flow of glucose-derived pyruvate into the TCA cycle^34,47^. The expression of PDHK-1 protein is significantly decreased by 0.49-, 0.68-, and 0.0007-fold after treatment with 1, 2, and 4 µM MB**8** in comparison to untreated cells, respectively (Fig. 3d). Our data suggest that MB**8** may play a role in inducing HepG2 cancer cell death by up-regulating the conversion of pyruvate to acetyl-CoA via the reduced HIF-1α expression-mediated suppression of PDHK-1 expression.

### Molecular bowl 8 and glucose transporter

Next, we used qRT-PCR to analyze the expression of glucose transporter-1 (GLUT-1) and glucose transporter type-4 (GLUT-4) mRNA in MB**8**-treated HepG2 cancer cells. The glucose transporter is a key rate-limiting factor in the transport and metabolism of glucose in cancer cells, which promotes higher glucose uptake to support increased cellular respiration. GLUT-1 is up-regulated in various malignant tumors, which depend on glycolysis for ATP generation^48^. GLUT-4 is up-regulated when the level of OXPHOS complex and the respiration rate increase^49–51^. None of the MB**8** concentrations showed a significant change in GLUT-1 mRNA expression compared to the untreated cells (Fig. 3e). However, the expression of GLUT-4 mRNA significantly increased by 1.8-fold when cells were treated with 4 µM MB**8**, in comparison to untreated cells (Fig. 3f). These findings suggest that MB**8** treatment predominantly involves acetyl-CoA and electron transport chain (ETC), followed by higher ROS production in HepG2 cancer cells.

### Molecular bowl 8 and ROS production by activated cytochrome c oxidase

**Figure 4:**
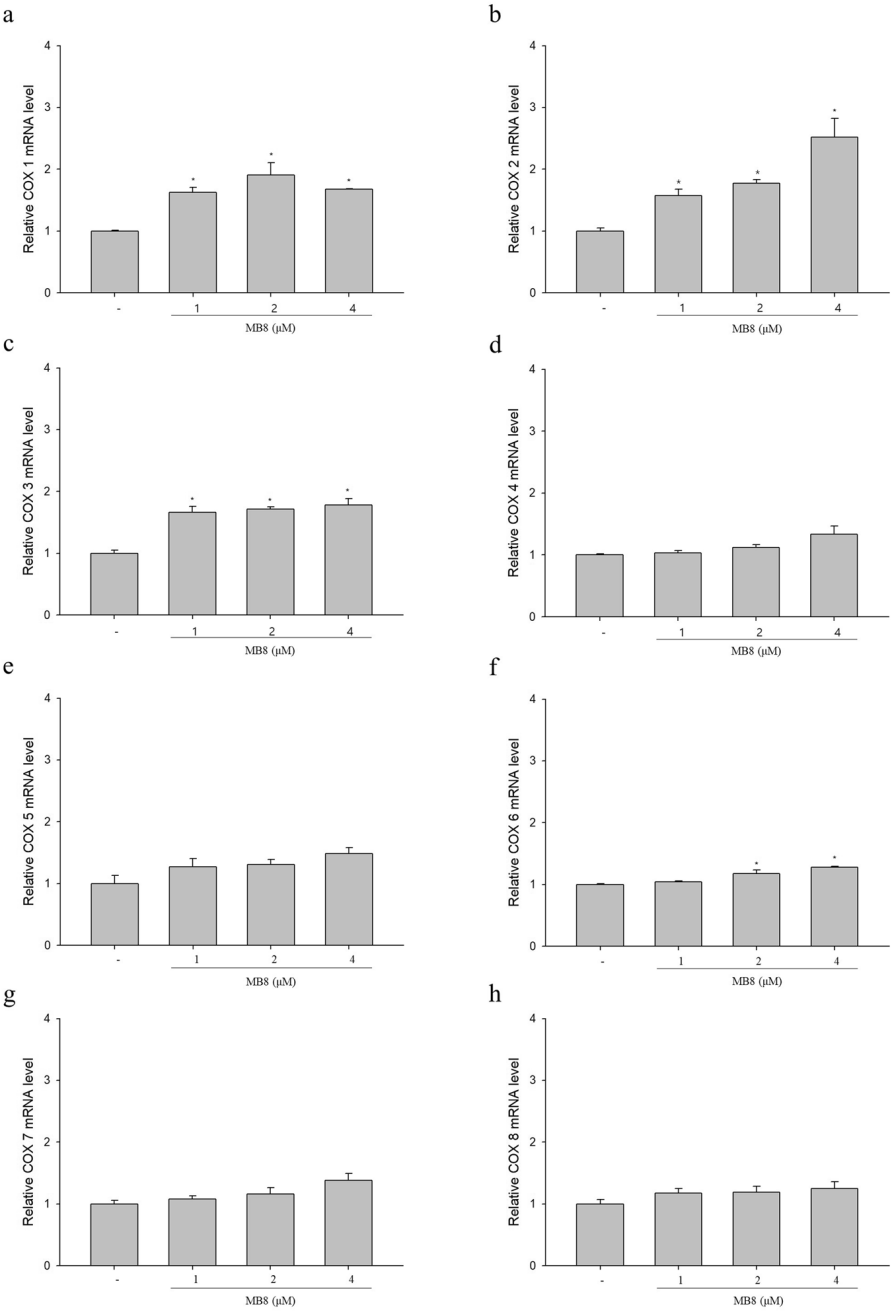
MB**8** activates electron transport system by increasing cytochrome c oxidase (COX) subunits. HepG2 cells (4 × 10^5^ cells/well) were seeded on a 6-well plate and incubated for 24 h. After that, MB8 was treated by concentration (1, 2 and 4 μM), and when it was 24 h, the level of mRNA expression was confirmed. (**a**–**h**) mRNA expression of COXs was detected by qRT-PCR. Representative data are shown as the mean ± standard error of the mean (SEM) of each group. *P < 0.05 compared with the control.

**Figure 5:**
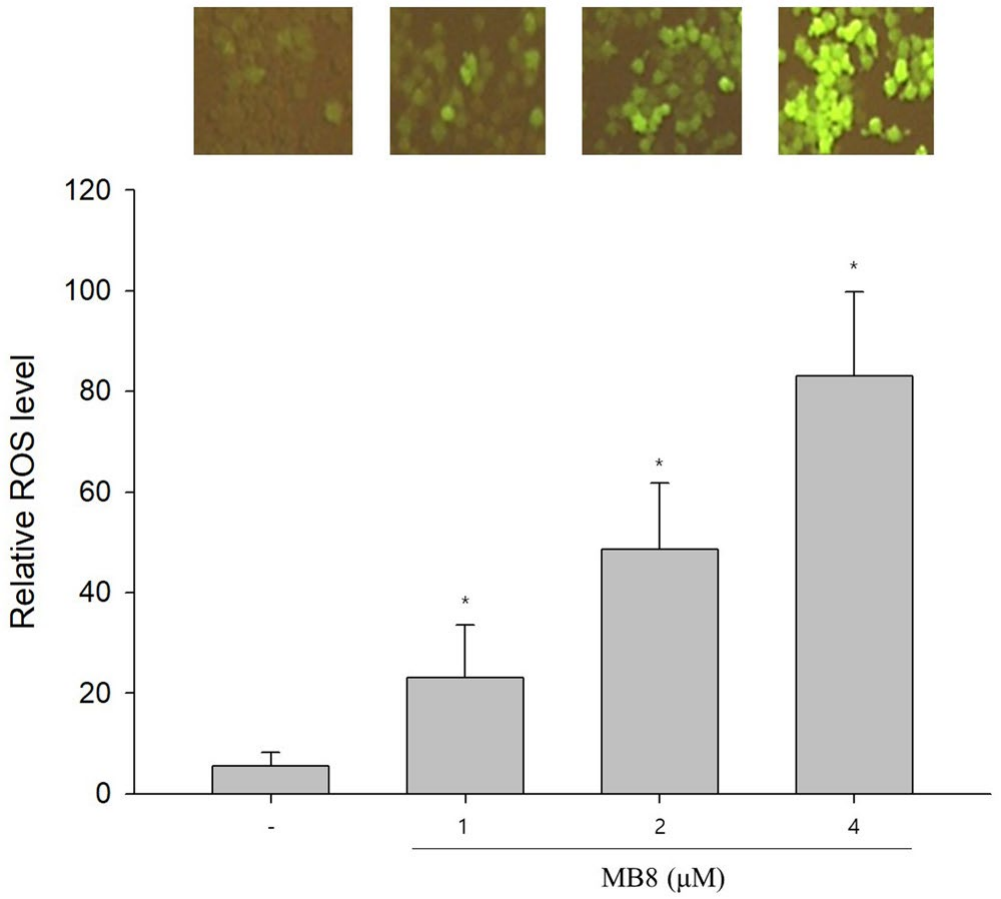
MB**8** induces ROS overexpression. HepG2 cells (2 × 10^5^ cells/well) were seeded on a 6-well plate and incubated for 24 h. After that, MB8 was treated by concentration (1, 2 and 4 μM), and when it was 24 h. The top panel shows microscopic images of DCF-DA fluorescence intensity that reflect the ROS level in HepG2 cells. Representative data are shown as the mean ± standard error of the mean (SEM) of each group. *P < 0.05 compared with the control.

To examine whether MB**8** regulates ETC-mediated ROS production, we analyzed the mRNA expression of cytochrome c oxidase subunit 1–8 and cellular ROS production. Expression of mRNA from the cytochrome c oxidase (COX) subunits (COX-1, 2, 3 and 6) significantly increased after treatment with MB**8** at all concentrations (1, 2 and 4 µM) in comparison to the untreated cells (Fig. 4). In particular, the mRNA expression of COX-2 showed dose-dependent increases of 1.6-, 1.8- and 2.5-fold for 1, 2 and 4 µM of MB**8**, respectively. In succession, MB**8** significantly increased ROS levels in a concentration-dependent manner to 4.2, 8.8 and 15.1 at concentrations of 1, 2 and 4 µM compared to the untreated cells (Fig. 5). COX, the terminal enzyme in the respiratory electron transport chain of mitochondria, is a large integral membrane protein and consists of 3 mtDNA-encoded and 10 genomic DNA-encoded subunits^48^. COX activity regulates the overall rates of mitochondrial respiration and electron transport, resulting in the production of superoxide (O_2_^−^) as a by-product and a pro-oxidant to promote ROS-mediated signaling pathways^52–54^. Our data provide evidence that MB**8** induces cancer cell death by increasing the ETC, followed by increasing the ROS production.

### Molecular bowl 8 and classical apoptosis

**Figure 6:**
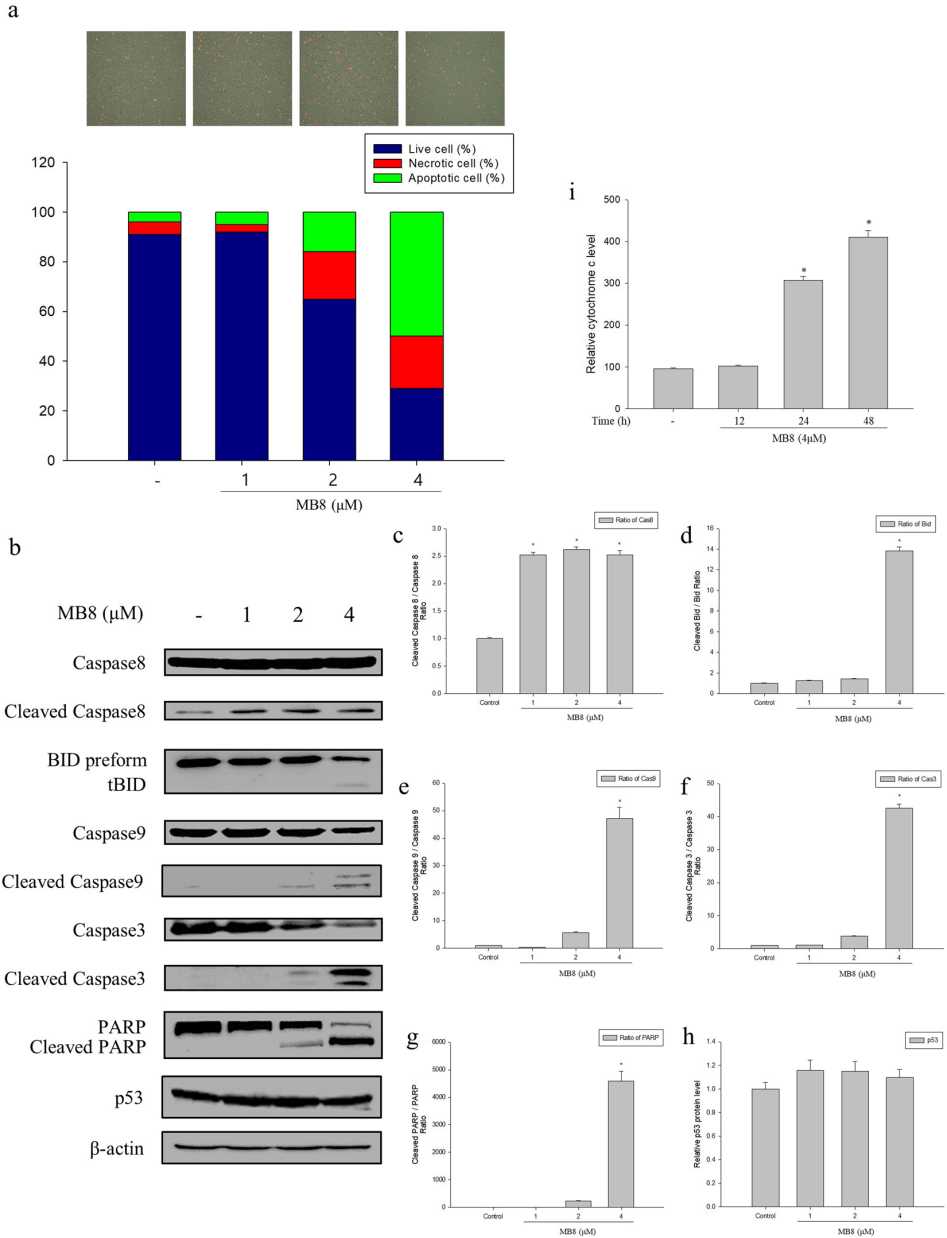
MB**8** induces apoptosis by activating the extrinsic and intrinsic pathway in HepG2 cells. (**a**) HepG2 cells (2 × 10^5^ cells/well) were seeded on a 6-well plate and incubated for 24 h. After that, MB8 was treated by concentration (1, 2 and 4 μM), and when it was 24 h. Apoptotic and necrotic cells were stained with green and red, respectively. (**b**) HepG2 cells (4 × 10^5^ cells/well) were seeded on a 6-well plate and incubated for 24 h. After that, MB8 was treated by concentration (1, 2 and 4 μM), and when it was 24 h. The protein levels were analyzed by Western blot. (**c**–**h**) Densitometric analysis of Western blots is represented as the mean band density. (i) HepG2 cells (2 × 10^5^ cells/well) were seeded on a 6-well plate and incubated for 24 h. After that, MB8 was treated by 4 μM, and when it was treated by times 12, 24 and 48 h. Cytochrome c levels were measured using cytochrome c ELISA kit. Representative data are shown as the mean ± standard error of the mean (SEM) of each group. *P < 0.05 compared with the control.

We determined whether the ROS-mediated cell death by MB**8** was apoptosis or necrosis. We confirmed that the number of HepG2 cells was decreased due to the cytotoxicity of MB**8** and more apoptotic cells were detected. Via quantification of the apoptotic cells and necrotic cells, we found the apoptotic cell population increased to 5%, 16%, and 50% at concentrations of 1, 2 and 4 µM MB**8**, respectively (Fig. 6a). These results show that MB**8** mediated HepG2 cancer cell death through apoptosis rather than necrosis. Next, we investigated the signal transduction pathway underlying the apoptosis-mediated cell death induced by MB**8** and found that it activated caspase-8 cleavage, and the ratio of cleaved caspase-8 and caspase-8 was significantly enhanced by 2.5-, 2.6-, and 2.5-fold at concentrations of 1, 2, and 4 µM of MB**8** compared with the untreated cells (Fig. 6c). In succession, the expression of Bid pre-form in the MB**8**-treated HepG2 cells significantly decreased in comparison with the untreated cells, while the expression of cleaved Bid was significantly induced. The ratio of cleaved Bid and Bid increased by 13.8-fold at a concentration of 4 µM compared to the untreated cells (Fig. 6d). The ratio of cleaved caspase-9 and caspase-9 also showed a significant increase of 47.2-fold at a concentration of 4 µM MB**8** compared to the untreated cells (Fig. 6e). Sequentially, MB**8** significantly induced the activation of caspase-3 and PARP by 42.5-fold and 4,581-fold, respectively, at a concentration of 4 µM in comparison with the untreated cells (Fig. 6f,g). As shown in Fig. 6h, there was no significant change in the expression of p53 protein after treatment with MB**8**. We also confirmed that MB**8** significantly increased cytochrome c release by 4.3-fold at a concentration of 4 µM in comparison with the untreated cells, and continued for 48 h (Fig. 6i). Based on these results, MB**8** induced HepG2 cancer cell death by activating both intrinsic and extrinsic apoptosis, followed by crosstalk between the intrinsic and extrinsic pathway through caspase-8 mediated cleavage of the Bcl-2 family member Bid, suggesting that the potential of cancer cell death by MB**8** was not associated with cell arrest via p53.

**Figure 7:**
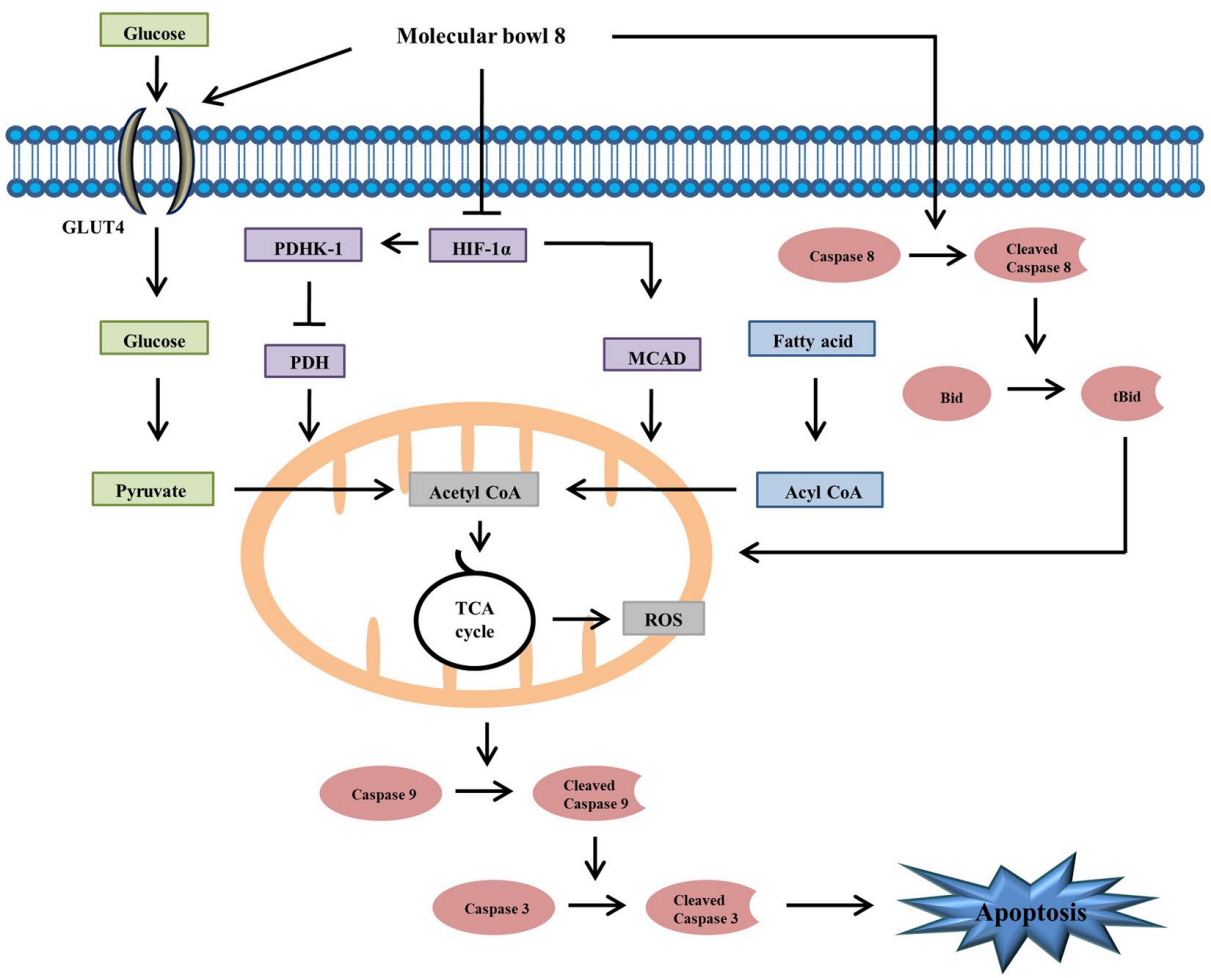
Schematic diagram showing the apoptosis effects of MB**8** in HepG2 cell. Increasing the expression of GLUT4 protein as well as promoting β-oxidation of fatty acids, thereby activating the TCA cycle in the mitochondria and increasing the production of ROS. These signal transduction activates the intrinsic apoptosis pathway, and cleavage of caspase 8 activates the extrinsic apoptosis pathway. Through these various pathways, MB8 induces strong apoptosis in HCC.

We have reported the synthesis and characterization of a new dipyridyl benzamide ligand and its subsequent coordination-driven self-assembly with Ru (II) *p*-cymene acceptors to obtain four molecular bowls. All of the new compounds have been characterized by ^1^H NMR^13^,C NMR, ESI-MS and the structure of the oxalate derived molecular bowl has been established by single-crystal X-ray analysis. The naphthalene derived molecular bowl facilitates cytotoxicity in HepG2 human HCC by HIF-1α-mediated cellular ROS production and glucose metabolism via 1) increased glycolysis through up-regulation of GLUT-4, 2) stimulated conversion of acetyl-CoA by suppression of PDHK 1, and 3) enhanced β-oxidation by stimulation of MCAD. Also, our *in vitro* experimental evaluations revealed that the MB**8** promotes apoptosis via crosstalk between intrinsic and extrinsic cell death pathways via Bid activation in HepG2 cancer cells (Fig. 7). We suggest that the MB**8** has development potential as a therapeutic agent. Further research should investigate the exact mechanisms by which the molecular bowl acts and assess the safety of the molecular bowl *in vivo* for further development as an anti-cancer therapeutic agent.

## Materials and Methods

### Materials

All chemicals used in this work were purchased from commercial sources. All solvents were distilled via standard methods prior to use. The starting arene−ruthenium acceptor clips were prepared as previously described^10,11^. The ^1^H and ^13^C NMR spectra were recorded with a Bruker 300 MHz spectrometer. Mass spectra for the self-assembled architectures were recorded using electrospray ionization with a MassLynx operating system at the Korea Basic Science Institute (Seoul, Korea). Elemental analyses were performed at the Technical Support Center, Pohang University of Science and Technology.

### Crystallographic data collection and structure refinement

The diffraction data from single crystals mounted on a loop were collected at 100 K on an ADSC Quantum 210 CCD diffractometer with synchrotron radiation (λ = 0.70000 Å) at Macromolecular Crystallography Beamline 2D, Pohang Accelerator Laboratory (PAL), Pohang, Korea. The raw data were processed and scaled using the HKL2000 program. The structure was solved by direct methods and the refinements were carried out with full-matrix least squares on F2 with appropriate software implemented in the SHELXTL program package. X-ray data for molecular bowl **6**: C_90_H_82_F_12_N_6_O_22_Ru_4_S_4_, M = 2360.14, triclinic, P, a = 12.712^3^ Å, b = 19.155^4^ Å, c = 20.243^4^ Å, α = 91.90^30^, β = 100.75^30^, γ = 90.95^30^, V = 4838.7^17^ Å^3^, Z = 2, T = 100 K, μ(synchrotron) = 0.753 mm^−1^, ρ_calcd_ = 1.620 Mgm^−3^, 11425 reflections measured, R_1_ = 0.1249 and wR_2_ = 0.3339 for 11425 reflections (I > 2σ(I)), R_1_ = 0.1851 and wR_2_ = 0.3681 (all data), GoF = 1.274, 1149 parameters, and 215 restraints. All of the non-hydrogen atoms were refined anisotropically. Hydrogen atoms were added to their geometrically ideal positions.

### Synthesis of dipyridyl benzamide donor 1

5-dibromobenzamide (177.1 mg, 0.635 mmol), 3-ethynylpyridine (183.4 mg, 1.78 mmol), CuI (5.0 mg, 0.026 mmol), [PdCl_2_(PPh_3_)_2_] (26.7 mg, 0.038 mmol), and PPh_3_ (10.0 mg, 0.038 mmol) were added to a round-bottom flask. THF (10 mL) and triethylamine (10 mL) were added to the flask and bubbled with N_2_ for 10 minutes. The resulting reaction mixture was refluxed under nitrogen for 2 days and cooled, after which the solvent was removed under vacuum. The resulting residue was purified by flash column chromatography (SiO_2_, ethylacetate with 2% triethylamine) to afford **1** as a white solid.

### General procedure for the self-assembly of molecular bowls 6–9

Benzamide donor **1** and arene-Ru(II) acceptor **2**, **3**, **4**, or **5** were stirred in 1.5 mL nitromethane/methanol (1:1) at room temperature for 6 h to obtain a clear solution, to which diethyl ether was added drop-wise to precipitate the product, which was washed twice with diethyl ether via centrifugation. The resulting crystalline powders were dried to obtain pure samples of molecular bowls.

### Cell culture

Human AGS, A549 and HCT-15 cells were cultured in RPMI-1640. SK-HEP-1 and HepG2 cells were cultured in Dulbecco’s modified essential medium (DMEM). All media were supplemented with 10% fetal bovine serum (FBS) and 1% penicillin-streptomycin in a 37 °C incubator with a 5% CO_2_ atmosphere.

### Cell viability assay

AGS, A549, HCT-15, SK-Hep-1 and HepG2 cells were seeded at a density of 1 × 10^5^ cells/mL in 96-well plates. 24 h later, the cells were treated with the different molecular bowls (molecular bowl **6**, **7**, **8**, **9** or donor and pre-cursor **2**, **3**, **4**, **5**) for 24 and 48 h. The cells were incubated with 5 mg/mL MTT [3-(4,5-dimethylthiazol-2-yl)-2,5-diphenyl-tetrazolium bromide (Sigma-Aldrich) for 4 h. After the supernatant was removed, 100 µL of DMSO per well was added to the cells and dissolved, and the formazan crystal formed using a shaker for 10 min. The optical density was measured at a 570 nm wavelength with a multi-reader (TECAN, Switzerland).

### GeneFishing

Total RNA from HepG2 cells was isolated with Trizol reagent (Invitrogen). The yield and purity of the RNA were checked by measuring the absorbance values at 260 nm and the ratio of 260 to 280 nm, respectively. First-strand cDNA was synthesized using the dT-ACP1 primer (GeneFishing^TM^ DEG Premix Kits, Seegene, Korea). Briefly, 2 µg of total RNA, and 2 µL dT-ATP (10 µM) were incubated at 80 °C for 3 min. Then, reverse transcription was performed for 1.5 h at 42 °C in a total volume of 20 µL containing total RNA, 4 µL of 5X first strand buffer, 5 µL of 2 mM dNTPs, 0.5 µL of RNase Inhibitor (40 U/µL), and 1 µL of M-MLV Reverse Transcriptase (200 U/µL). After adding 80 µL of RNase-free water, second-strand cDNA synthesis with random PCR amplification was performed using dT-ACP2 and 20 arbitrary ACPs (GeneFishing^TM^ DEG Premix Kits, Seegene, Korea) as primers. The second-strand cDNA synthesis was performed in a total volume of 20 µL containing 3 µL of first-strand cDNA, 2 µL arbitrary dT-ACP2 primer, 1 µL of dT-ACP2, and 10 µL of 2X Master Mix. The PCR conditions for second-strand synthesis consisted of 94 °C for 5 min, 50 °C for 3 min, and 72 °C for 1 min at one cycle. Next, 40 cycles of amplification were performed with denaturation at 95 °C (40 sec), annealing at 65 °C (40 sec), extension at 72 °C (40 sec), and final extension step at 72 °C (5 min).

### Tali^®^ image-based cytometric assay

Apoptosis was measured with the Tali^®^ Image-Based Cytometer (Thermo Fisher Scientific, Waltham, MA, USA). HepG2 cells were seeded at a density of 2 × 10^5^ cells/mL/2 mL in 6-well plates. Then, cells were treated with different concentrations of MB**8**, ranging from 1 µM to 4 µM for 24 h. Cells were harvested using Trypsin/EDTA reagent, and stained using the Tali^®^ Apoptosis Kit. For the determination of apoptotic cells, cells were stained using the annexin V-Alexa Flour^®^ 488 conjugate and necrotic cells were estimated by propidium iodide (PI) staining. The population was assessed using the Tali cytometer.

### Quantitative Real-Time PCR

Total RNA from HepG2 cells was isolated with Trizol reagent. The yield, and purity of the RNA were checked by measuring the absorbance values at 260 nm and the ratio of 260 to 280 nm, respectively. Then, 2 µg of total RNA in a 20 µL volume was transcribed using the PrimeScriptII 1st strand cDNA Synthesis kit (Takara, Japan). Quantitative real-time polymerase chain reactions (qRT-PCR) were performed with a MX3005P (Stratagene, USA) using the following primers (Table 2). For real-time PCR, SYBR Premix Ex Taq II (Takara, Japan) was used. The final volume of the reaction mixture was 25 µL containing 2 µL cDNA template, 12.5 µL master mix, 1 µL each primer (10 µM stock solution), and 9.5 µL sterile distilled water. The thermal cycling profile consisted of a pre-incubation step at 95 °C for 10 min, followed by 40 cycles at 95 °C (30 s), 55 °C (60 s) and 72 °C (30 s). Relative quantitative evaluation of SCAD, MCAD, LCAD, VLCAD, GLUT-1, -4, COX-1 to 8 was performed using comparative cycle threshold (CT).

**Table 2:** The primer sequences used for real-time PCR.

Gene name	Primer sequences
SCAD	5′- CGGCAGTTACACACCATCTAC-3′ (forward)
	5′- GCAATGGGAAACAACTCCTTCTC-3′ (reverse)
MCAD	5′- GGAAGCAGATACCCCAGGAAT-3′ (forward)
	5′- AGCTCCGTCACCAATTAAAACAT-3′ (reverse)
LCAD	5′- TGCAATAGCAATGACAGAGCC-3′ (forward)
	5′- CGCAACTACAATCACAACATCAC-3′ (reverse)
VLCAD	5′- TCAGAGCATCGGTTTCAAAGG-3′ (forward)
	5′- AGGGCTCGGTTAGACAGAAAG-3′ (reverse)
GLUT-1	5′- CCATCCACCACACTCACCAC-3′ (forward)
	5′- GCCCAGGATCAGCATCTCAA-3′ (reverse)
GLUT-4	5′- AGAGTCTAAAGCGCCT-3′ (forward)
	5′- CCGAGACCAACGTGAA-3′ (reverse)
COX-1	5′- GGCCTGACTGGCATTGTATT-3′ (forward)
	5′- TGGCGTAGGTTTGGTCTAGG-3′ (reverse)
COX-2	5′- ACAGACGAGGTCAACGATCC-3′ (forward)
	5′- TCGATTGTCAACGTCAAGGA-3′ (reverse)
COX-3	5′- CCCGCTAAATCCCCTAGAAG-3′ (forward)
	5′- ATGGTGAAGGGAGACTCGAA-3′ (reverse)
COX-4	5′- GCCCATGTCAAGCACCTGTC-3′ (forward)
	5′- CCCTGTTCATCTCAGCAAAGCTC-3′ (reverse)
COX-5	5′- GATGCGCTCCATGGCATCT-3′ (forward)
	5′- TCTTTGCAGCCAGCATGATCTC-3′ (reverse)
COX 6	5′- GGCTGTAGCATTCGTGCTATCC-3′ (forward)
	5′- TCTGAAAGATACCAGCCTTCCTCA-3′ (reverse)
COX 7	5′- AAAGCGCACTAAATCGTCTCC-3′ (forward)
	5′- CATTCTATTCCGACTTGTGTTGCTA-3′ (reverse)
COX 8	5′- TGTACTCCGTGCCATCATGT-3′ (forward)
	5′- TCACGAAGCAGGAGGTAAGC-3′ (reverse)
β-actin	5′-TCACCCACACTGTGCCCATCTACGA-3′ (forward)
	5′-GGATGCCACAGGATTCCATACCCA-3′ (reverse)

### Western blot

Cells were lysed with PRO-PREP^TM^ Protein Extraction for 1 h. Lysates were collected after centrifugation at 12,000 × g for 10 min at 4 °C. Equal amounts of protein measured by bovine serum albumin (BSA) were boiled for 5 min, and fractionated by SDS-PAGE. Primary antibodies against the following proteins were used: MCAD (Proteintech); HIF-1α, PDHK-1, Caspase8, BID, Caspase9, Caspase3, PARP and p53 (Cell Signaling Technology; CST). HRP-conjugated anti-mouse and anti-rabbit (CST) secondary antibody were used. In all Western blotting experiments, the blots were re-probed with anti-β-actin antibody to ensure equal protein loading. Bands were visualized with the EZ-Western Lumi Pico reagents according to the manufacturer’s instructions.

### Intracellular ROS measurement

Intracellular ROS were analyzed with dichlorofluorescein diacetate (DCF-DA). HepG2 cells were harvested and incubated with 100 mM DCF-DA (dissolved in DMSO) for 30 min at 37 °C. Then, the HepG2 cells were washed three times with PBS, and the fluorescence of DCF-DA was detected by a multi-reader with excitation at 485 nm and emission at 530 nm.

### Cytochrome c measurement

To determine cytochrome c release, we used the ELISA kit (Enzo Life Science). Briefly, HepG2 cells were treated with MB8 4 µM for 12, 24 and 48 h. HepG2 cells were harvested after centrifugation, resuspended with permeabilization buffer, vortexed and then incubated on ice for 5 min and centrifuged. The supernatants, which contained the cytosolic fraction of cytochrome c, were retained, and RIPA and cell lysis buffer 2 were used to resuspend the pellet. The lysate was incubated on ice for 5 min. After centrifugation, the supernatant containing the mitochondrial fraction of cytochrome c was saved and measured by a multi-reader at 405 nm.

### Statistics

All data are presented as the mean ± standard error of the mean (SEM). The 50% inhibitory concentrations (IC_50_) was calculated by analyzing the log of the concentration-response curves by nonlinear regression analysis. The results were analyzed for statistically significant experimental differences by one-way analysis of variance (ANOVA) and post-hoc Duncan’s multiple range tests (DMRT). Statistical analysis was done with SPSS software (version 12.0). A *P* < 0.05 was considered statistically significant.

## Electronic supplementary material

Supplementary Information

## Data Availability

The datasets generated during and/or analysed during the current study are not publicly available due to patent registration but are available from the corresponding author on reasonable request.
