# Supplementary material for: New Self-assembled Supramolecular Bowls as Potent Anticancer Agents for Human Hepatocellular Carcinoma
Source: Sci Rep. 2019 Jan 18;9:242. doi: 10.1038/s41598-018-36755-9 (PMC6338755; doi:10.1038/s41598-018-36755-9)
Supplement: Supplementary file 1 — Supplementary Information [file 41598_2018_36755_MOESM1_ESM.docx]

**Supplementary Information**

**New Self-assembled Supramolecular Bowls as Potent Anticancer Agents for Human Hepatocellular Carcinoma**

Hae Seong Song^1#^ Young Ho Song^2#^, Nem Singh^2^, Hyunuk Kim^3^, Hyelin Jeon^1^, Inhye Kim^1*^, Se Chan Kang^1*^ and Ki-Whan Chi^2*^

^1^Department of Oriental Medicine Biotechnology, College of Life Sciences, Kyung Hee University, Yongin 17104, Republic of Korea

^2^Department of Chemistry, University of Ulsan, Ulsan 44610, Republic of Korea

^3^Convergence Materials Laboratory, Korea Institute of Energy Research, Daejeon 28119, Republic of Korea.

^#^Hae Seong Song and Young Ho Song contributed equally to this work

**Table of Contents**

| S.N. | Contents | Page |
| --- | --- | --- |
| 1. | ^1^H, ^13^C NMR and ESI-MS spectra of **1** and **6 - 9** | S2-S7 |
| 2. | X-ray crystal structures of **6** | S8 |
| 3. | X-ray crystal structure parameters of molecular bowl **6** | S9 |
| 4. | Selected bond lengths [Å] and angles [°] for molecular bowl **6**. | S10 |


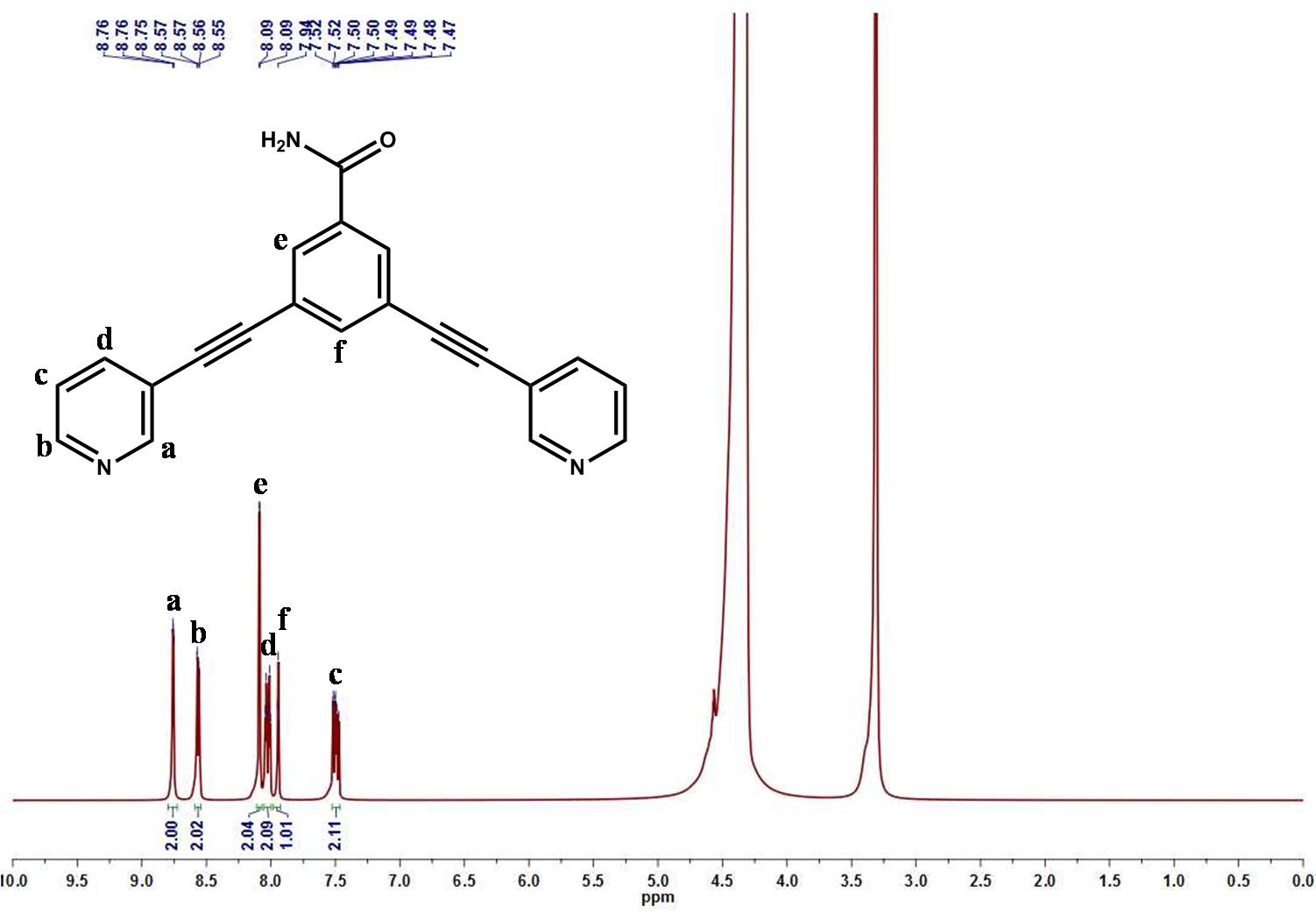


**Figure S1:** ^1^H NMR spectrum of benzamide donor **1** in CD_3_OD/CD_3_NO_2_.


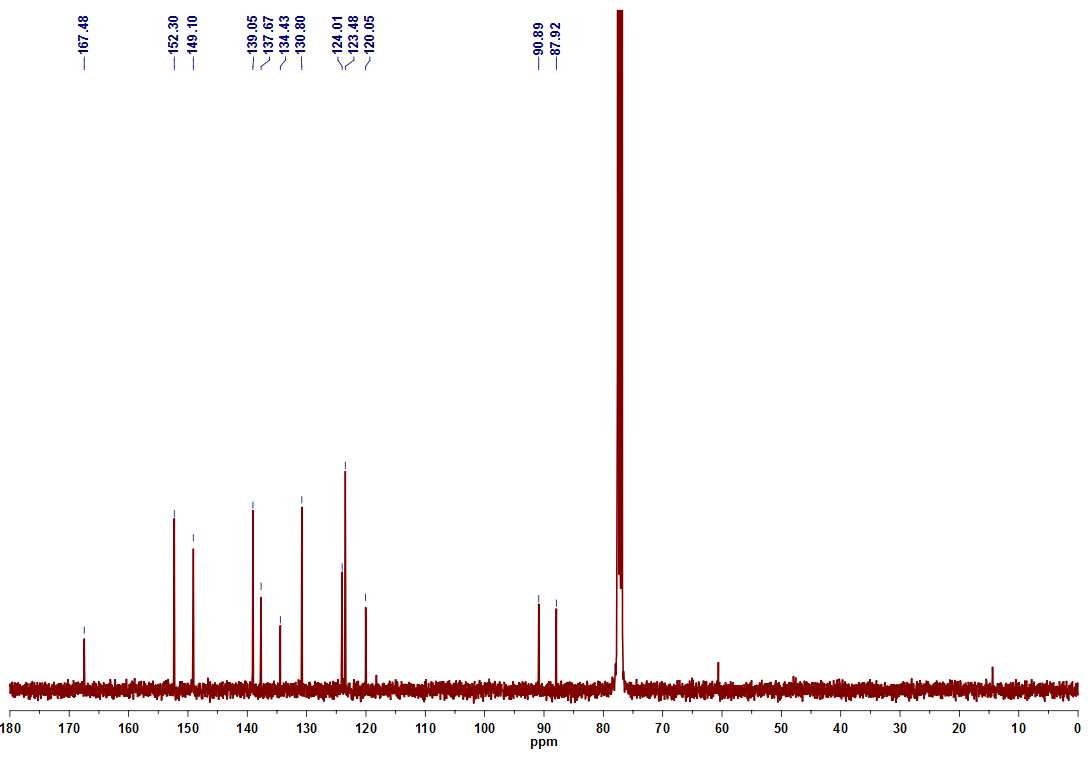


**Figure S2:** ^13^C NMR spectrum of benzamide donor **1** in CDCl_3_.


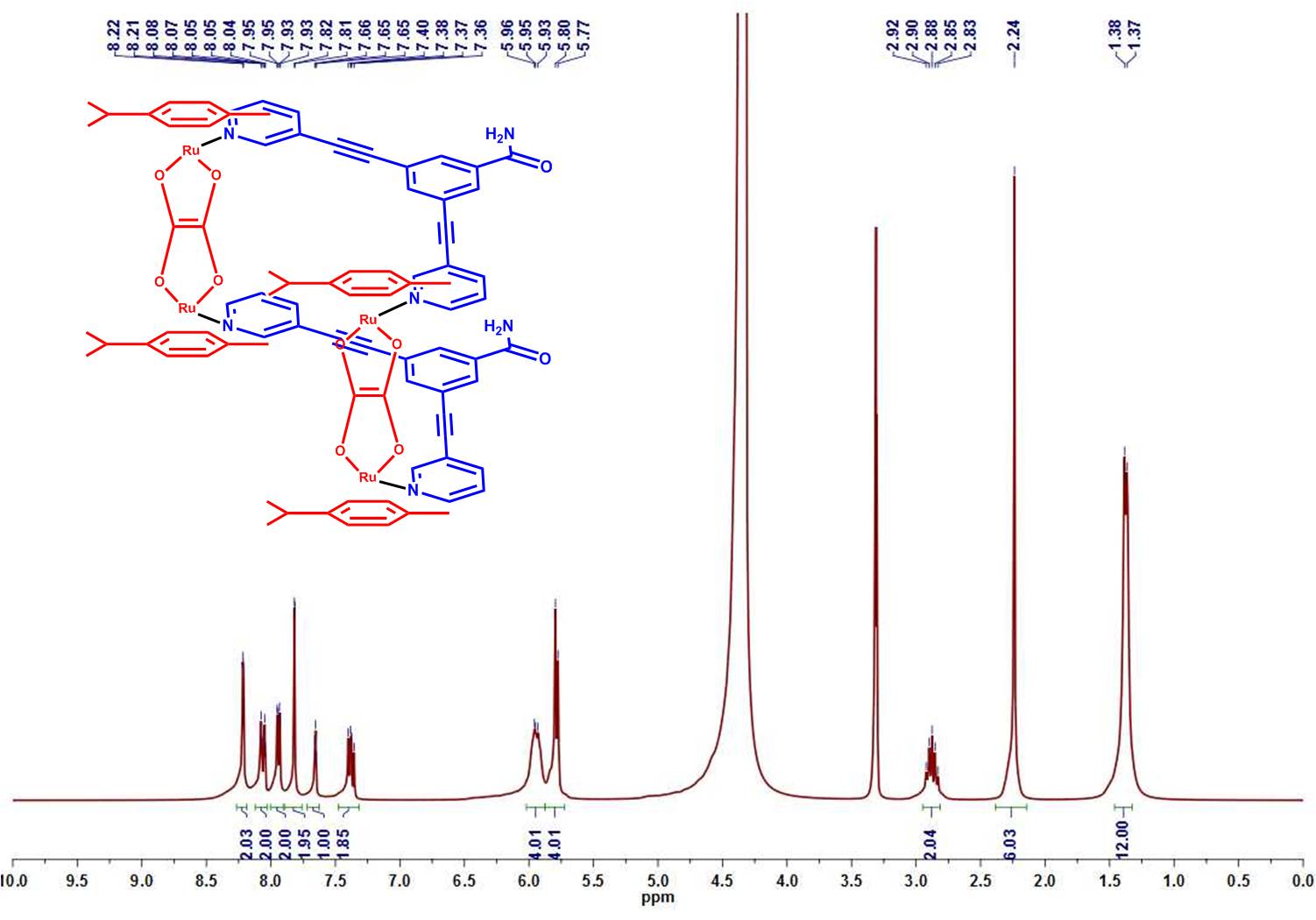


**Figure S3:** ^1^H NMR spectrum of molecular bowl **6**.


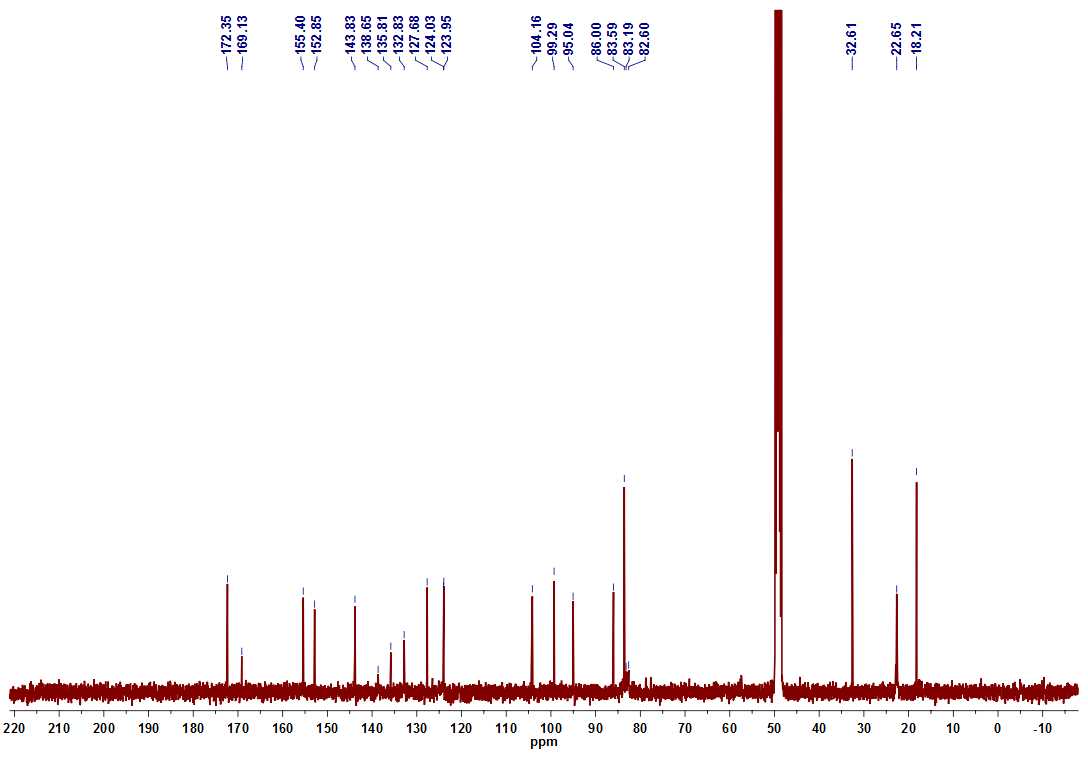


**Figure S4:** ^13^C NMR spectrum of molecular bowl **6**.


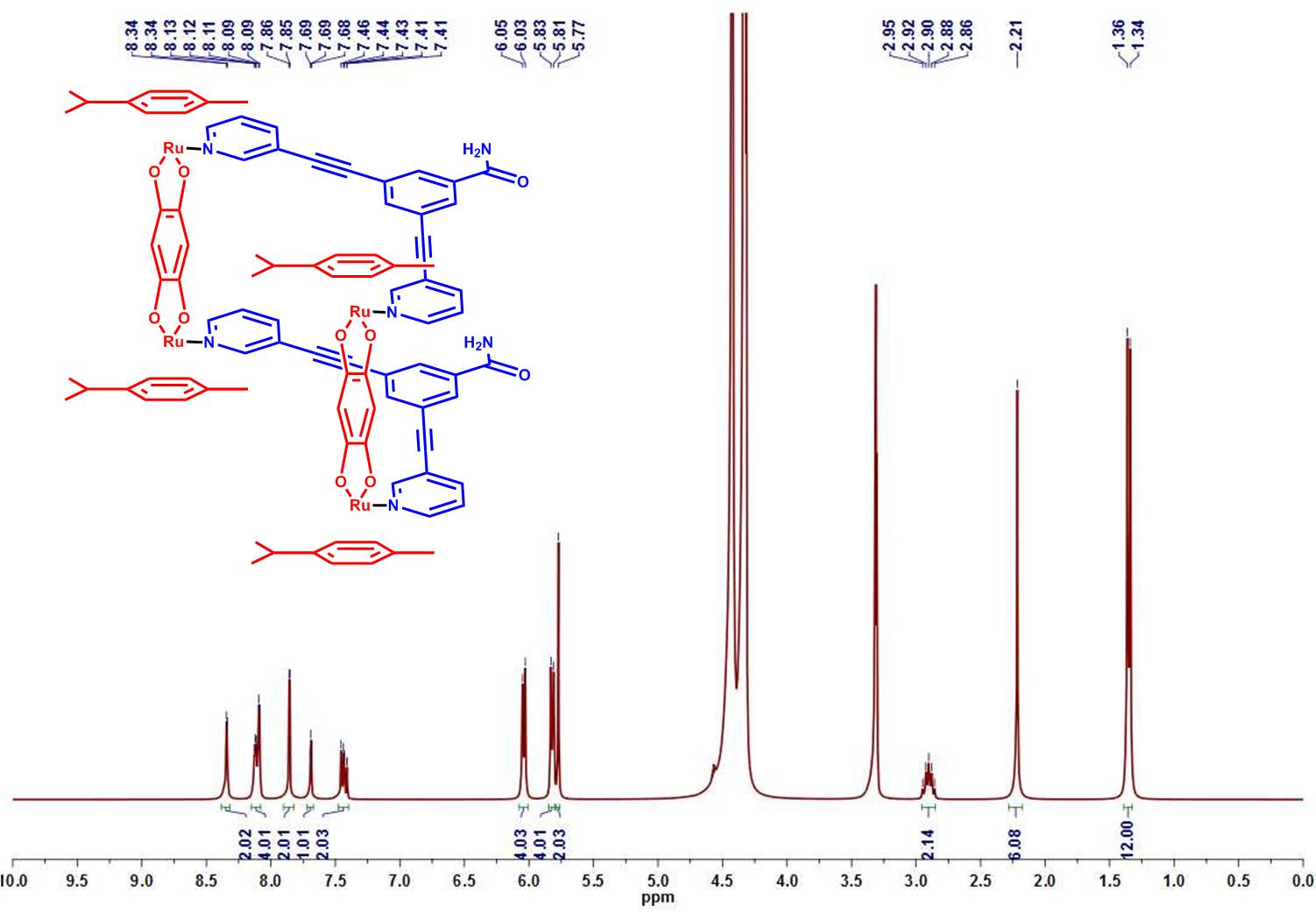


**Figure S5:** ^1^H NMR spectrum of molecular bowl **7**.


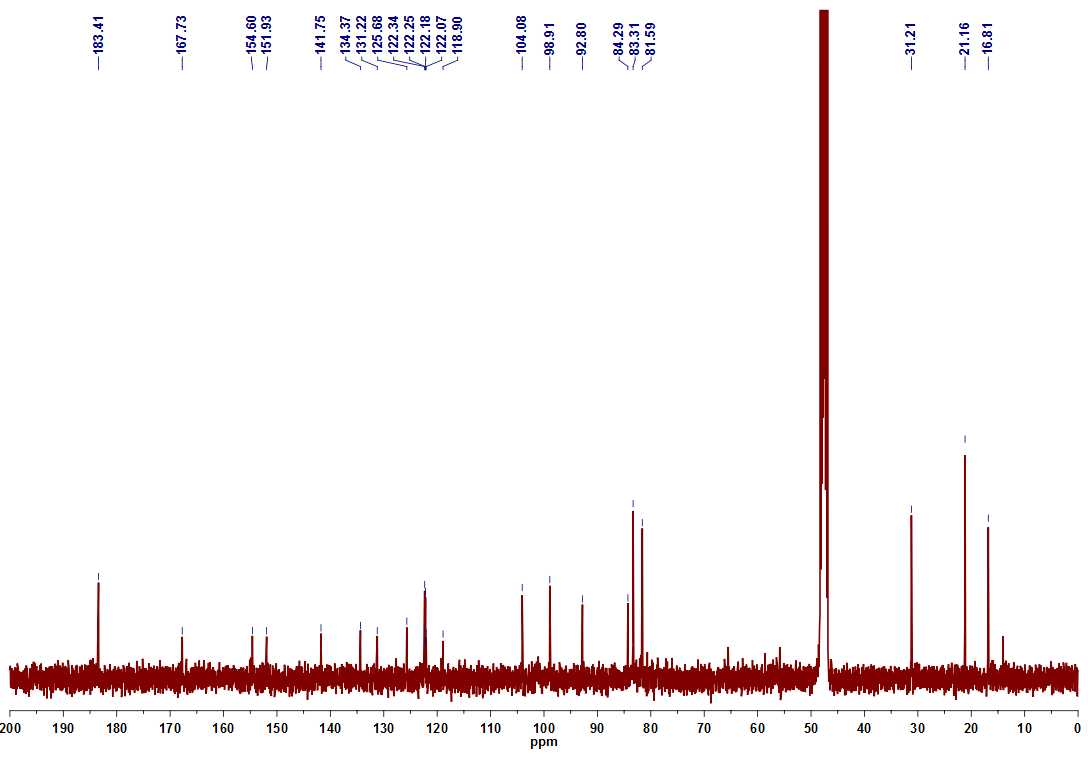


**Figure S6:** ^13^C NMR spectrum of molecular bowl **7**.


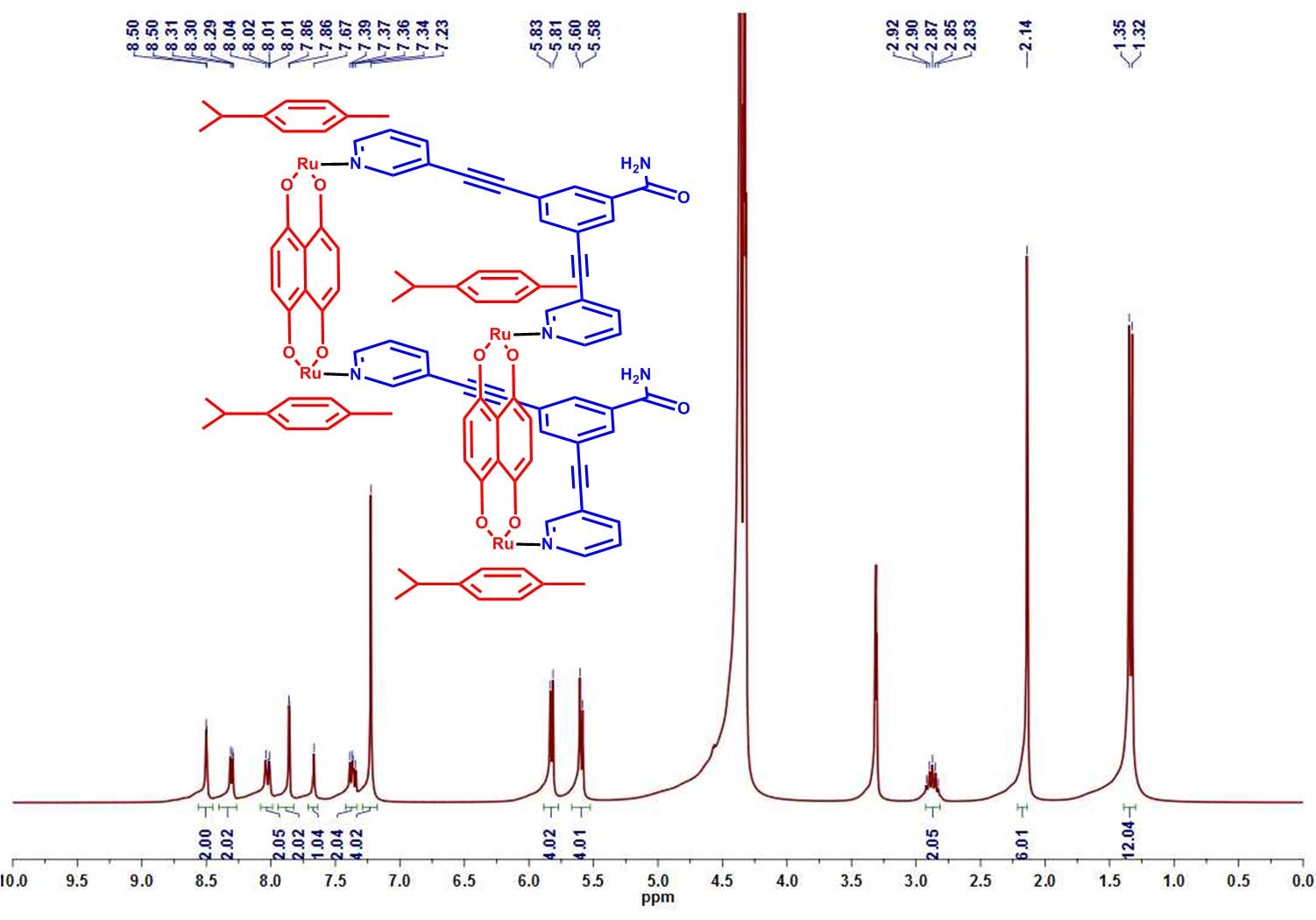


**Figure S7:** ^1^H NMR spectrum of molecular bowl **8**.


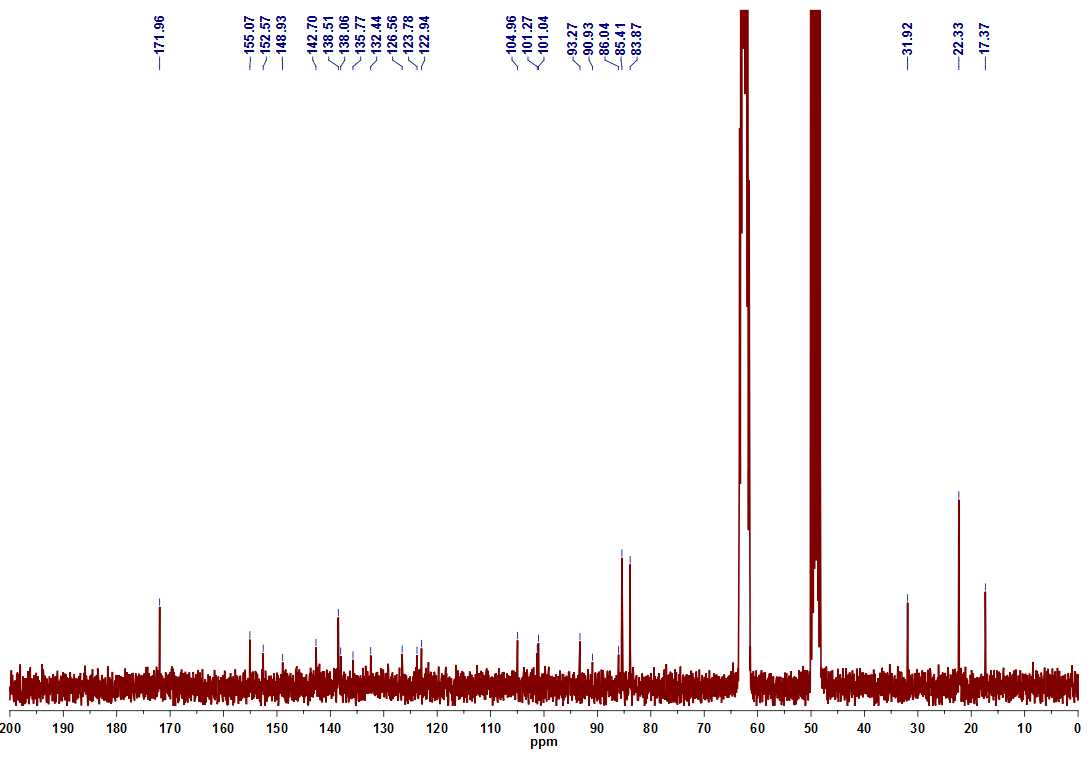


**Figure S8:** ^13^C NMR spectrum of molecular bowl **8**.
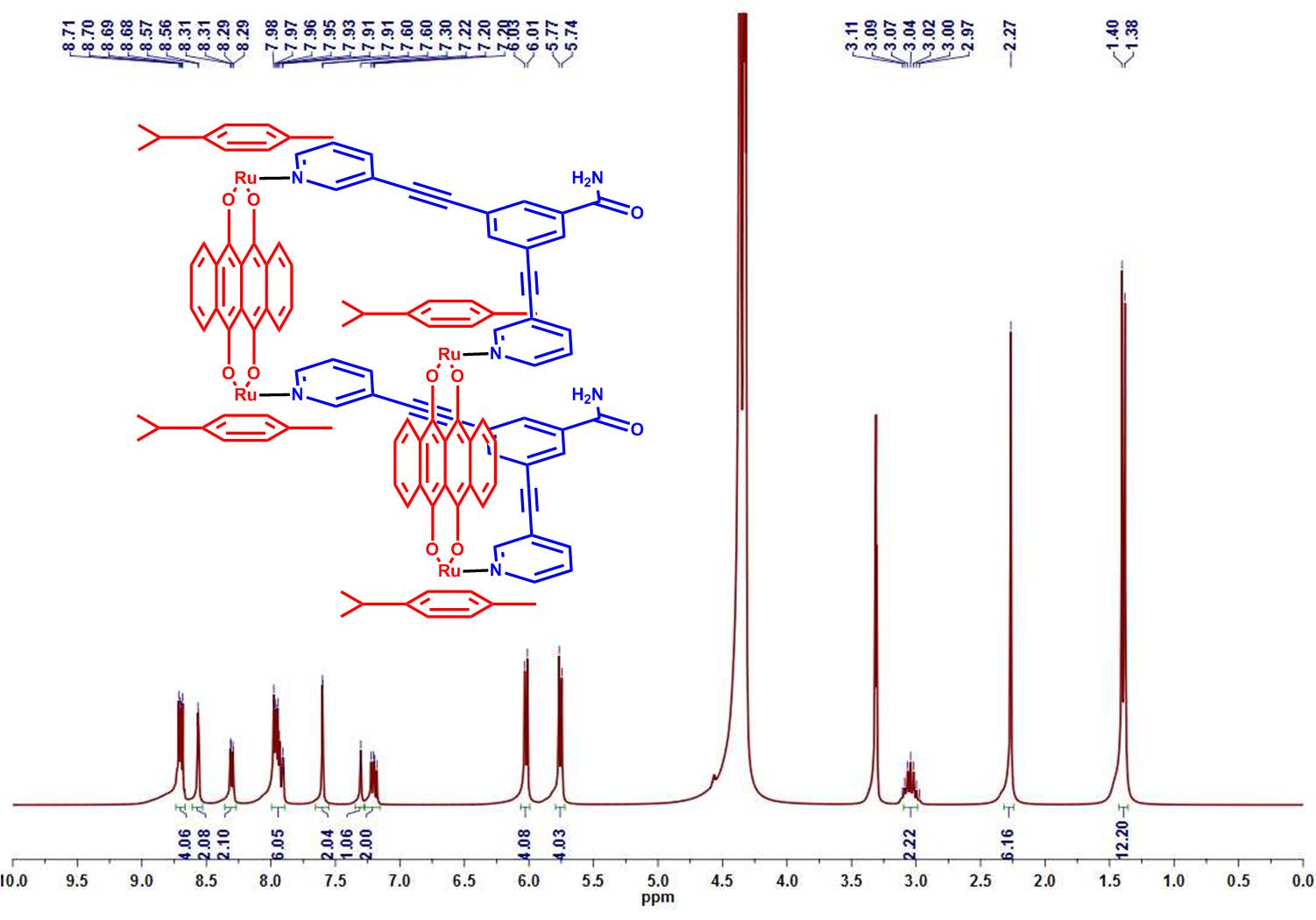


**Figure S9:** ^1^H NMR spectrum of molecular bowl **9**.


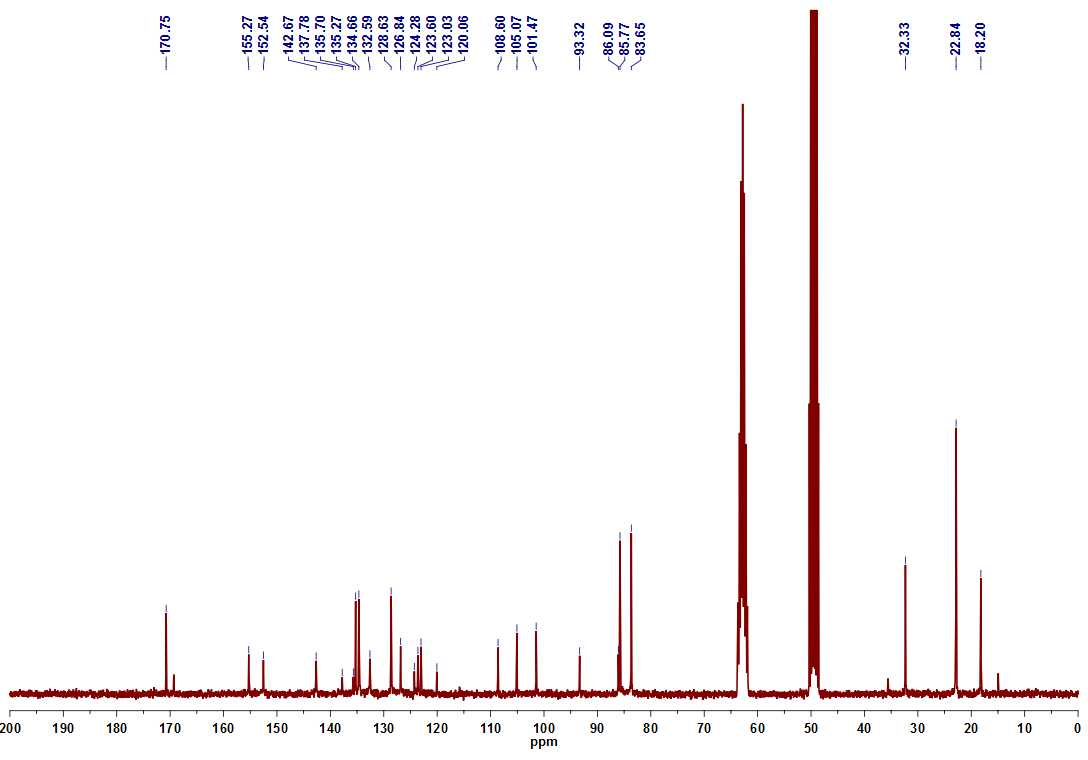


**Figure S10:** ^13^C NMR spectrum of molecular bowl **9**.


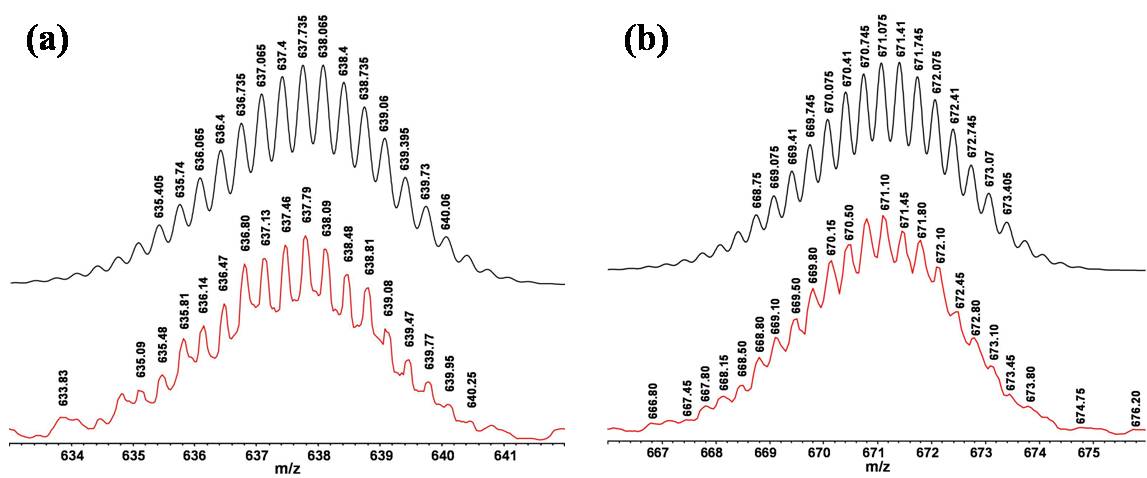

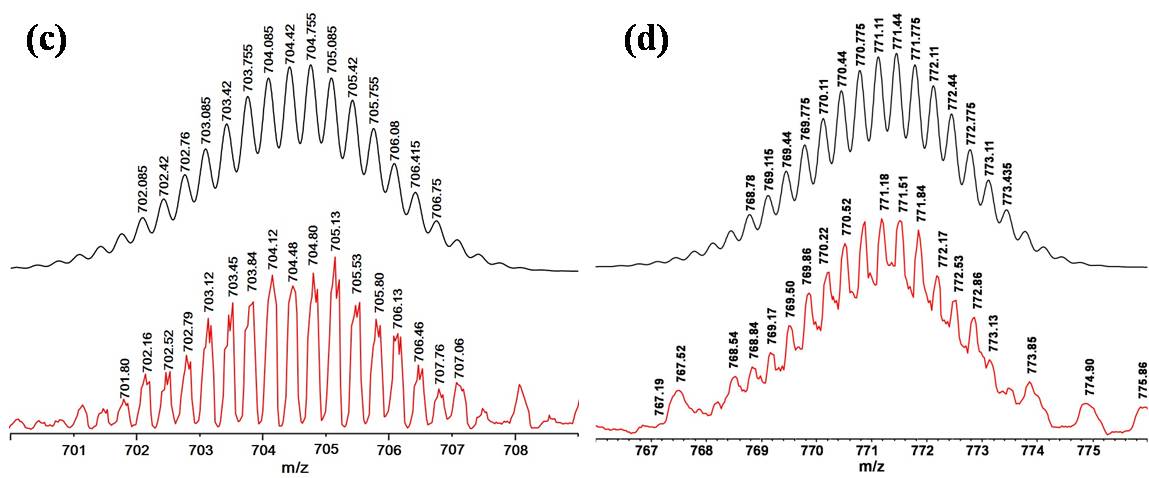


**Figure S11:** Electrospray ionization–mass spectrometry spectra of molecular bowls (A) [**6**-3OTf ]^3+^, (B) [**7**-3OTf ]^3+^, (C) [**8**-3OTf ]^3+^ and (d) [**9**-3OTf ]^3+^.


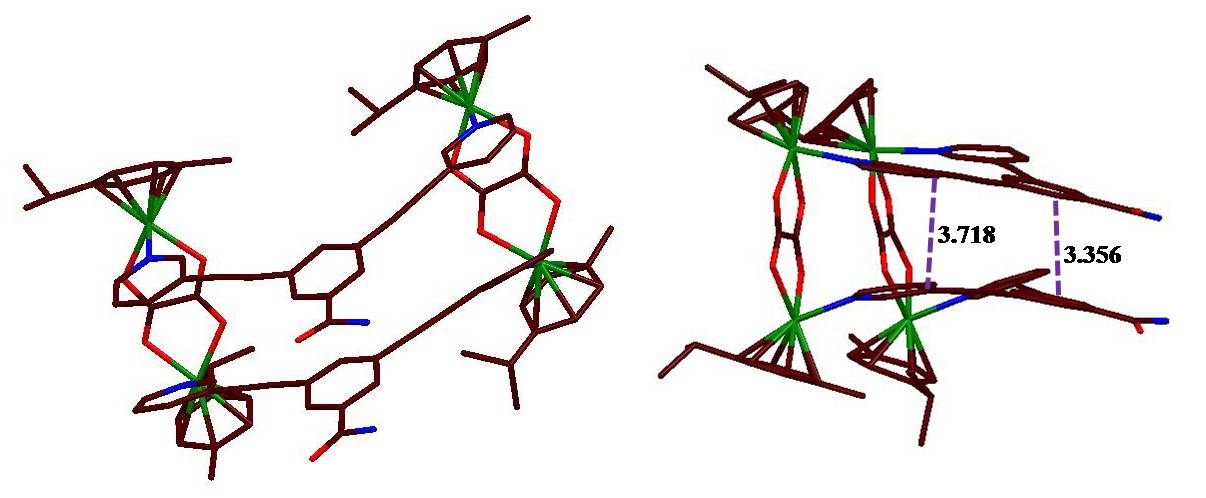


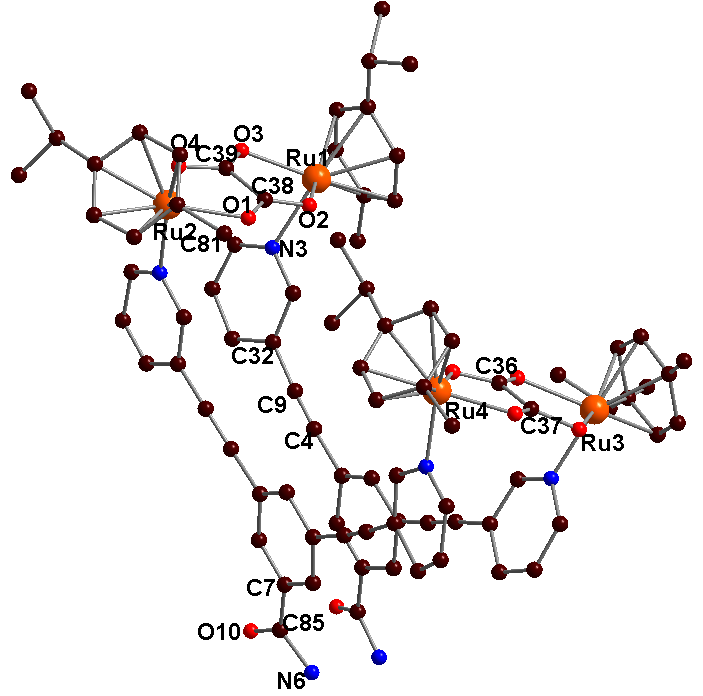


**Figure S12:** X-ray crystal structure of molecular bowl **6** presented in stick model (top) and ball stick model (bottom).

**Table S1:** Crystal data and structure refinement for molecular bowl **6**.

Identification code p-1_sqd

Empirical formula C_90_ H_82_ F_12_ N_6_ O_22_ Ru_4_ S_4_

Formula weight 2360.14

Temperature 100(2) K

Wavelength 0.70000 Å

Crystal system Triclinic

Space group P-1

Unit cell dimensions a = 12.712(3) Å α= 91.90(3)°.

b = 19.155(4) Å β= 100.75(3)°.

c = 20.243(4) Å γ = 90.95(3)°.

Volume 4838.7(17) Å3

Z 2

Density (calculated) 1.620 Mg/m3

Absorption coefficient 0.753 mm-1

F(000) 2376

Crystal size 0.40 x 0.20 x 0.20 mm3

Theta range for data collection 2.57 to 22.88°.

Index ranges -14<=h<=14, -21<=k<=21, -22<=l<=22

Reflections collected 21875

Independent reflections 11425 [R(int) = 0.0538]

Completeness to theta = 22.88° 82.1 %

Absorption correction Semi-empirical from equivalents

Max. and min. transmission 0.8640 and 0.7527

Refinement method Full-matrix least-squares on F2

Data / restraints / parameters 11425 / 215 / 1149

Goodness-of-fit on F2 1.274

Final R indices [I>2sigma(I)] R1 = 0.1249, wR2 = 0.3339

R indices (all data) R1 = 0.1851, wR2 = 0.3681

Largest diff. peak and hole 2.232 and -1.314 e.Å-3

##

**Table S2:** Selected bond lengths [Å] and angles [°] for molecular bowl **6**.

| Ru(1)-O(2) 2.113(11)  Ru(1)-O(3) 2.115(11)  Ru(1)-N(3) 2.156(15)  Ru(3)-O(5) 2.091(16)  Ru(3)-O(6) 2.136(13)  Ru(3)-N(1) 2.129(15)  C(4)-C(9) 1.14(3)  C(9)-C(32) 1.43(3)  C(7)-C(85) 1.451(18)  O(10)-C(85) 1.20(3)  N(6)-C(85) 1.40(3)  O(9)-C(84)-N(5) 123.3(18)  O(9)-C(84)-C(5) 121(2)  N(5)-C(84)-C(5) 116(2)  C(4)-C(9)-C(32) 176(2)  O(2)-C(38)-O(1) 125.4(17)  O(2)-C(38)-C(39) 117.7(16)  O(1)-C(38)-C(39) 116.9(16)  C(38)-O(2)-Ru(1) 112.7(11) | Ru(2)-O(1) 2.111(11)  Ru(2)-O(4) 2.121(10)  Ru(2)-N(4) 2.106(15)  Ru(4)-O(8) 2.080(18)  Ru(4)-O(7) 2.110(13)  Ru(4)-N(2) 2.09(2)  C(36)-C(37) 1.47(3)  C(38)-C(39) 1.51(2)  C(5)-C(84) 1.454(17)  O(9)-C(84) 1.24(3)  N(5)-C(84) 1.35(3)  O(10)-C(85)-N(6) 122.9(19)  O(10)-C(85)-C(7) 122(3)  N(6)-C(85)-C(7) 115(3)  O(3)-C(39)-O(4) 125.7(16)  O(3)-C(39)-C(38) 117.6(15)  O(4)-C(39)-C(38) 116.4(15)  O(2)-Ru(1)-O(3) 77.5(4)  O(2)-Ru(1)-N(3) 85.2(5) |
| --- | --- |

_____________________________________________________________

Symmetry transformations used to generate equivalent atoms
